# IL-33 expression in response to SARS-CoV-2 correlates with seropositivity in COVID-19 convalescent individuals

**DOI:** 10.1038/s41467-021-22449-w

**Published:** 2021-04-09

**Authors:** Michal A. Stanczak, David E. Sanin, Petya Apostolova, Gabriele Nerz, Dimitrios Lampaki, Maike Hofmann, Daniel Steinmann, Marvin Krohn-Grimberghe, Robert Thimme, Gerhard Mittler, Cornelius F. Waller, Edward J. Pearce, Erika L. Pearce

**Affiliations:** 1 Department of Immunometabolism, Max Planck Institute of Immunobiology and Epigenetics, Freiburg, Germany; 2 Proteomics, Max Planck Institute of Immunobiology and Epigenetics, Freiburg, Germany; 3 Department of Medicine II, Medical Center-University of Freiburg, Faculty of Medicine, University of Freiburg, Freiburg, Germany; 4 Occupational Medical Service, Medical Center-University of Freiburg, Faculty of Medicine, University of Freiburg, Freiburg, Germany; 5 Department of Cardiology and Angiology I, University Heart Center Freiburg, Freiburg, Germany; 6 Department of Medicine I, Medical Center-University of Freiburg, Faculty of Medicine, University of Freiburg, Freiburg, Germany; 7 Faculty of Biology, University of Freiburg, Freiburg, Germany; 8 Present Address: The Bloomberg-Kimmel Institute for Cancer Immunotherapy at Johns Hopkins, Baltimore, MD USA

**Keywords:** Interleukins, SARS-CoV-2, Epidemiology

## Abstract

Our understanding of severe acute respiratory syndrome coronavirus 2 (SARS-CoV-2) is still developing. We perform an observational study to investigate seroprevalence and immune responses in subjects professionally exposed to SARS-CoV-2 and their family members (155 individuals; ages 5–79 years). Seropositivity for SARS-CoV-2 Spike glycoprotein aligns with PCR results that confirm the previous infection. Anti-Spike IgG/IgM titers remain high 60 days post-infection and do not strongly associate with symptoms, except for fever. We analyze PBMCs from a subset of seropositive and seronegative adults. TLR7 agonist-activation reveals an increased population of IL-6^+^TNF^-^IL-1β^+^ monocytes, while SARS-CoV-2 peptide stimulation elicits IL-33, IL-6, IFNa2, and IL-23 expression in seropositive individuals. IL-33 correlates with CD4^+^ T cell activation in PBMCs from convalescent subjects and is likely due to T cell-mediated effects on IL-33-producing cells. IL-33 is associated with pulmonary infection and chronic diseases like asthma and COPD, but its role in COVID-19 is unknown. Analysis of published scRNAseq data of bronchoalveolar lavage fluid (BALF) from patients with mild to severe COVID-19 reveals a population of IL-33-producing cells that increases with the disease. Together these findings show that IL-33 production is linked to SARS-CoV-2 infection and warrant further investigation of IL-33 in COVID-19 pathogenesis and immunity.

## Abstract (web summary)

Our understanding of the immune response to SARS-CoV-2 infection is still incomplete. Here, the authors find that IL-33, produced during immune recall potentially by CD14^+^ monocytes, correlates with CD4^+^ T cell activation, anti-SARS-CoV-2 antibody titer, and disease severity in a cohort of convalescent individuals professionally exposed to the virus.

## Introduction

SARS-CoV-2 represents a unique public health challenge in modern times, and as of February 2021 more than 110,000,000 individuals have been infected worldwide (World Health Organization data). Several studies have shown that SARS-CoV-2 infected individuals seroconvert and develop neutralizing antibodies that are important for protective immunity^1–3^. In addition to the humoral immune response, other work has indicated that both CD4^+^ and CD8^+^ T cells with SARS-CoV-2 reactivity are present within the blood of convalescent individuals^4–6^.

During acute infection, pro-inflammatory cytokines can contribute to the pathology of SARS-CoV-2-induced acute respiratory disease syndrome, the life-threatening form of this infection. For instance, high serum IL-6, IL-8, TNF, and other cytokines at the time of hospitalization correlated with patient survival^7,8^. Increased cytokine levels were also detected in patients admitted to intensive care units as opposed to less symptomatic individuals^9^. Nevertheless, cytokine levels in SARS-CoV-2 patients appear to be lower than in patients with cytokine release syndrome, sepsis, or influenza^10,11^. Little is known about persisting cytokine signatures in convalescent individuals and how these might relate to humoral and cellular immune responses.

In this study, we set out to investigate seroprevalence and immune responses in subjects professionally exposed to SARS-CoV-2 and their family members (155 individuals; ages 5–79 years). We show that after recovery from COVID-19, individuals have persisting, circulating PBMCs that produce IL-33 in response to virus-specific T cell activation, which correlates with seropositivity. To the best of our knowledge, this is a completely novel finding in the context of COVID-19 and warrants further investigation.

## Results and discussion

**Fig. 1:**
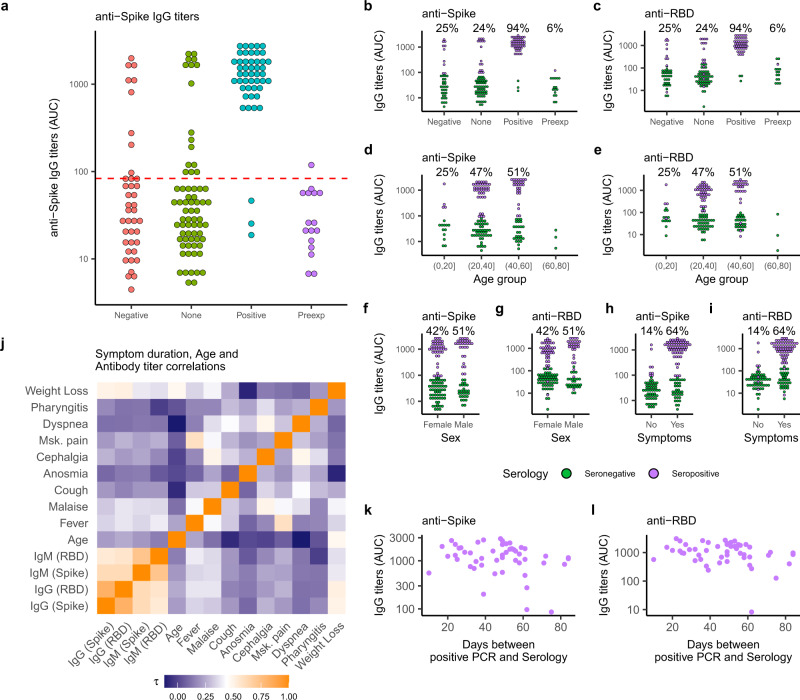
Serological characterization of the study population. **a** Anti-Spike IgG titers shown as area under titration curve (AUC) in sera from investigated subjects grouped by SARS-CoV-2 PCR status (Negative-red circles, None-green circles, Positive-blue circles) plus pre-pandemic historical sera (Preexp, magenta circles). The dotted line illustrates the estimated threshold above which subjects were classified as “Seropositive” (mean + 2xSD–red). Mean and SD was calculated for all IgG titers below the maximum value measured from Preexp sera (*n* = 63). **b**–**i** Anti-Spike (**b**, **d**, **f**, **h**) and anti-RBD (**c**, **e**, **g**, **i**) IgG titers shown as AUC for investigated subjects grouped by SARS-CoV-2 PCR status (**b**–**c**), age group (**d**–**e**), sex (**f**–**g**) and reported symptoms (**h**–**i**). Subjects are colored by anti-Spike IgG serology results (seropositive-purple circles, seronegative-green circles). Percentage seropositive subjects within each category are indicated. **j** Detected antibody titers from seropositive subjects were correlated to each other, symptom duration, and subject age using a Kendall rank correlation. A heatmap of the Kendall rank correlation coefficient ($\tau$) is shown for each pair of variables. **k**–**l**, Anti-Spike (**k**), and anti-RBD (**l**) IgG titers shown as a function of time for seropositive subjects. The approximate time of infection was taken as the day of positive SARS-CoV-2 PCR status.

We took the opportunity to study a cohort of individuals with a known professional exposure, within a relatively small timeframe (~2–3 months), as the pandemic was developing. We assessed viral transmission among these individuals and their family members. Specifically, we used an ELISA to measure IgG and IgM titers against the SARS-CoV-2 spike glycoprotein^12^. Our study population included 155 subjects who either tested positive for SARS-CoV-2 via PCR or were in contact with SARS-CoV-2-infected individuals. Demographic and disease characteristics of the study population are indicated in Supplementary Table 1. The median age was 36 years and 98 (63.2%) were females; 99 (64.5%) had professional exposure to the virus, 44 (28.4%) were exposed within their household and 12 subjects had either multiple or different/unknown exposure sources. Symptoms of upper respiratory tract infection within the last 16 weeks prior to blood collection were reported by 97 (62.6%) individuals. SARS-CoV-2 PCR testing was performed on 87 individuals, with 47 (30.3%) having a positive PCR result. We evaluated the presence and abundance of antibodies against the spike protein of SARS-CoV-2 in the serum of the study population at a single time point, as well as in 16 historical sera collected prior to the onset of the COVID-19 pandemic. We found the highest antibody titers among individuals with a positive SARS-CoV-2 PCR (Fig. 1a), as well as several cases where subjects with a negative PCR result possessed antibodies against the viral protein (9/40). As the date of the PCR and the collection of serum samples were separated by a median time of 49 days, it is possible that these individuals became infected after a PCR was carried out. In addition, we discovered few cases (3/47) where a positive PCR result did not associate with elevated anti-Spike IgG titers.

We used the anti-Spike IgG signal measured in pre-exposure sera to set a threshold to define seropositive cases (Fig. 1a–red dashed line). This resulted in 70 seropositive individuals. We then probed collected sera for IgG and IgM antibodies against the spike protein and its receptor-binding domain (RBD) finding a robust correlation between these measurements (Fig. 1b, c, j, and Supplementary Fig. 1a–e). Seropositive individuals in our cohort were predominantly between 20–60 years of age (Fig. 1e, f), and were less frequent among 0–20-year-olds (4/16) (Fig. 1e, f), despite documented exposure to infected individuals within their families. We found no association between sex and antibody titers (Fig. 1f, g). Most seropositive subjects reported experiencing symptoms (Fig. 1h). Eight out of 70 subjects who had high anti-Spike IgG titers were asymptomatic (Fig. 1h), although anti-RBD IgG titers in some of these individuals fell below the positive threshold (Fig. 1i). To explore possible links between clinical features and antibody titers, we calculated Kendall rank correlation coefficients ($\tau$) between symptom duration, subject age, and antibody titers in pairs (Fig. 1j). Beyond the strong correlation between IgG and IgM titers for both Spike and RBD, we found modest to no correlation among clinical features. To further explore these associations, we fitted logistic regression models to reported symptoms among seropositive subjects incorporating age, sex, and time between positive PCR and serology as independent variables, setting the symptom report (yes = 1, no = 0) as the outcome predicted by the antibody titer. Within our cohort, elevated antibody titers against Spike and RBD were reasonable predictors of experiencing fever (Supplementary Fig. 1h–k), but not for other symptoms. These results might suggest that people with strong immune responses against the virus feel sicker during acute infection, but also have persistent IgM/IgG antibodies as a result of this stronger antiviral response. While previous work^1,13^ has found higher IgM/IgG titers in patients with more severe disease, recent studies highlight the importance of antibody kinetics as a predictor of disease progression^14,15^. It is possible that the comparatively late time-point of serum sampling, as well as the mild disease severity in our cohort, could explain the discrepancy between those findings and our results. Finally, we observed no appreciable drop in antibody titers in the study population, even 60 days after active infection (Fig. 1k–l and Supplementary Fig. 1f–g).

**Fig. 2:**
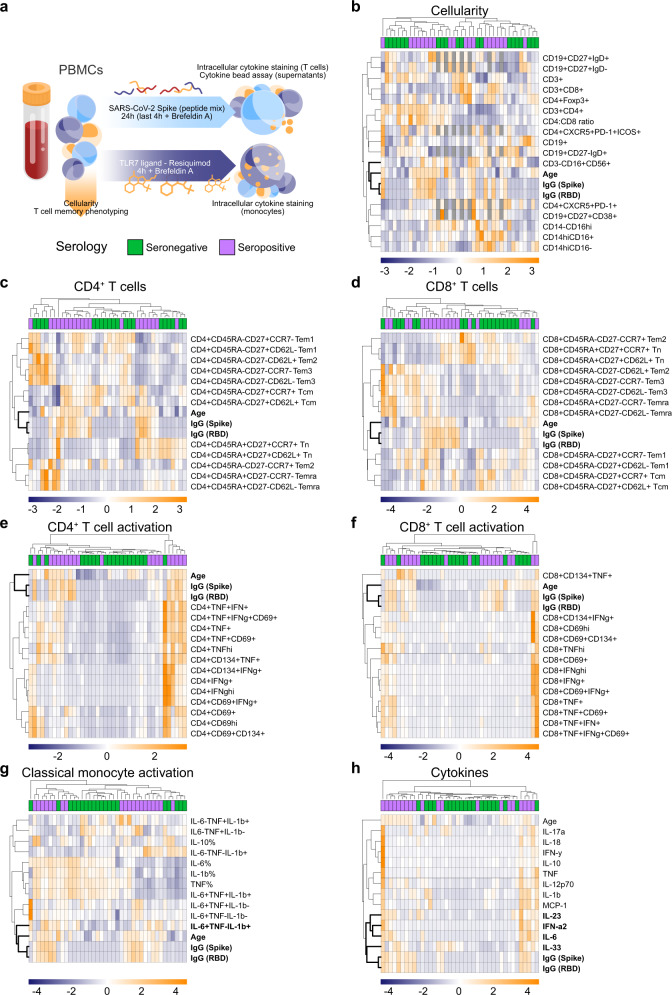
PBMCs from convalescent and non-infected subjects differ only in innate cytokine production profile. **a**–**h** PBMCs in blood collected from a subset of 20 seropositive and 20 seronegative subjects were isolated, analyzed via flow cytometry for cellular composition (**b**–**d**), and stimulated with a SARS-CoV-2 spike peptide mix or vehicle control (**e**–**f**, **h**), or with TLR7 agonist Resiquimod or vehicle control (**g**) for indicated times (**a**). Activation of CD3^+^CD4^+^ (**e**), CD3^+^CD8^+^ (**f**) and CD14^hi^CD16^−^ cells (**g**) plus cytokine secretion into culture media (**h**) were measured via flow cytometry. Control corrected (treatment–control) scaled data (where the mean was subtracted from each value then divided by the standard deviation) was then visualized as heatmaps. Scaled anti-Spike and anti-RBD IgG titers plus subject age were included in the results. Data were clustered based on Euclidean distances between features (rows) or subjects (columns), with clusters indicated with dendrograms. Serology results are included for each subject (seropositive–purple; seronegative–green). The closest associations to antibody titers are highlighted in bold letters. Missing values are shown in dark gray.

SARS-CoV-2 represents a unique public health challenge in modern times, with subsequent waves^2^ of infection being an active area of research. While antibodies can neutralize the virus and are therefore likely to be important for protective immunity^2^, it is probable that the pathologic effects of SARS-CoV-2 infection are cell-mediated. The development of IgG antibodies during infection is an indication that a CD4^+^ T follicular helper cell response is mounted against this virus^16,17^, and recent work has indicated that both CD4^+^ and CD8^+^ T cells capable of making IFN-γ and TNF are present within the blood of convalescent patients^3,6,17^. We therefore decided to look broadly at cellular immune responses in convalescent patients. We focused on both SARS-CoV-2 Spike protein-specific T cell responses and innate monocyte responses and measured cytokines known to be produced by both adaptive and innate immune cells. We did this for a subset of 20 convalescent individuals in our cohort, comparing them to 20 seronegative subjects in our study (Supp. Table 2). For all individuals, we assessed a single time point after convalescence. We purified PBMCs from these individuals and examined their composition (Fig. 2a–d) and activation following stimulation (Fig. 2a, e–h), correlating these results to antibody titers (Fig. 2 in bold) using conventional markers (Supplementary Fig. 2) and 2 different stimuli (Fig. 2a). Overall, we did not detect a strong association between the cellular composition, immune cell activation, time from active infection, and antibody titers, (Fig. 2 and Supplementary Fig. 3) with the latter more closely associated with subject age than any other measured parameter (Fig. 2b–f). However, upon examination of TLR7-driven classical monocyte activation (Fig. 2g), we discovered that the percentage of IL-6^+^TNF^-^IL-1β^+^ monocytes clustered closely with IgG titers. Moreover, SARS-CoV-2 peptide-induced production of IL-33, IL-6, IFN-ɑ2, and IL-23 in these cultures (Fig. 2h) also clustered together with IgG titers. Critically, IL-33 production was more closely related to IgG titers than any other measured parameter (Supplementary Fig. 3), including subject age.

**Fig. 3:**
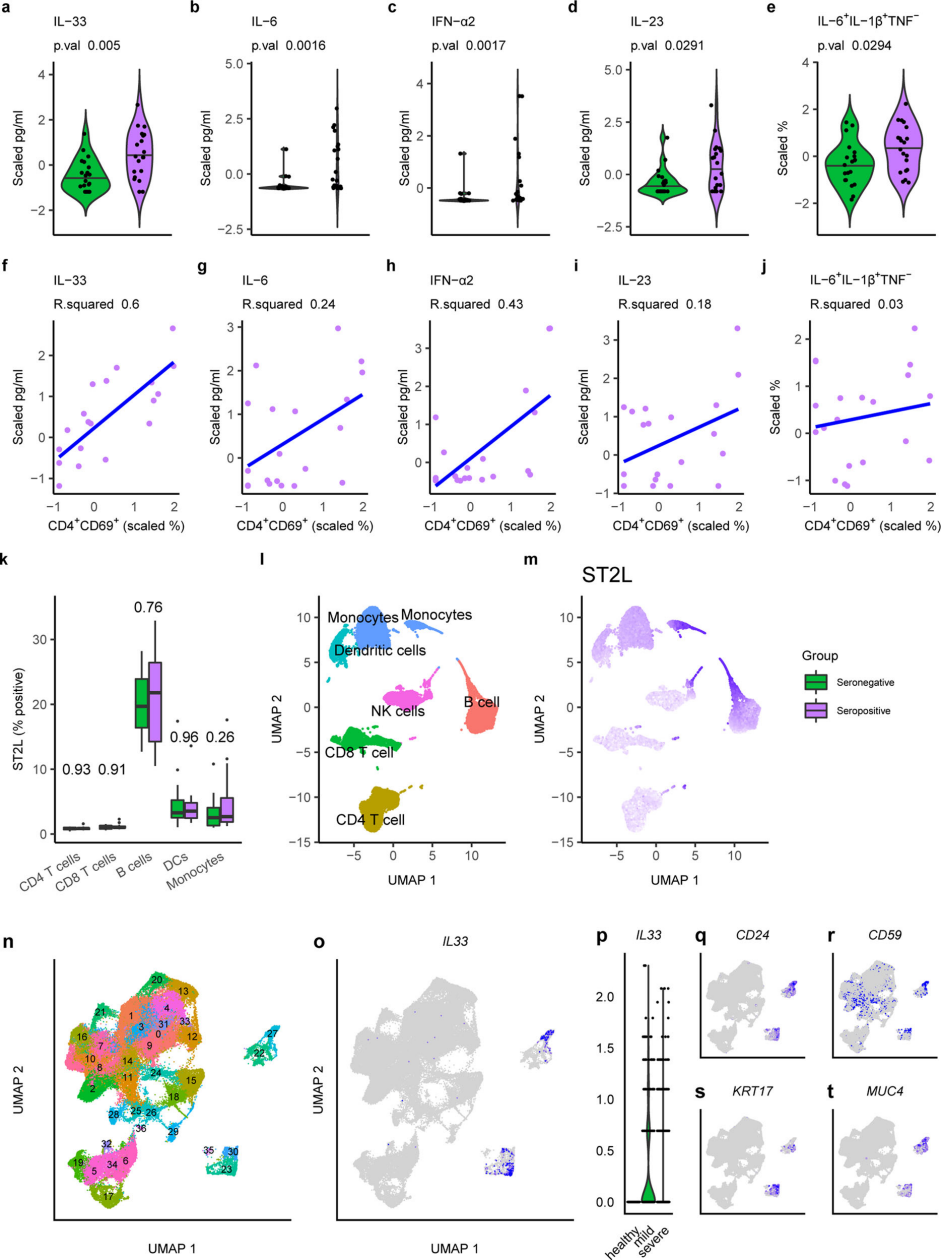
IL-33 production correlates with T cell activation and disease severity in SARS-CoV-2 infected subjects. **a**–**e** Cytokine production measured in the media of cultured PBMCs (**a**–**d**) or via intracellular staining of CD14^hi^CD16^−^ monocytes from seropositive (purple) and seronegative (green) subjects are shown as scaled values. Two-tailed Mann–Whitney *U* tests were calculated and the resulting *p*-value is reported. Values for subjects are shown as dots, the sample distribution is presented as a violin plot and the population median is a black line. **f**–**j** Scaled cytokine production versus corresponding CD4 T cell activation (CD4^+^CD69^+^) in PBMC cultures of seropositive individuals are shown. Linear regression curves fitted to these data (blue) alongside *R*^2^ values are provided. **k**–**m** ST2L expression as a percentage of positive cells was measured in the indicated cell populations via flow cytometry in PBMCs from seropositive (purple) and seronegative (green) subjects (*n* = 40). Two-tailed Mann–Whitney *U* tests were calculated and resulting *p* values are reported. Boxplots indicating the mean (black line), 25th and 75th quantiles (edges), and interquartile ranges (whiskers), as well as outliers (dots), are shown. Flow cytometry staining is presented in a dimensional reduction projection (UMAP), showing immune cell clusters (**l**) and ST2L median fluorescence intensity (MFI) (**m**). Scaled MFI is indicated on a gradient from low (gray) to high (purple). **n**–**t** Single-cell RNA sequencing data from the bronchoalveolar lavage fluid of 3 healthy individuals compared to 9 SARS-CoV-2 infected subjects with different disease severities (3 milds; 6 severe) was retrieved from a public database (GSE145926), grouped into cell clusters (**n**) and analyzed for the expression of IL-33 (**o**). Relative expression levels are indicated on a gradient from low (light gray) to high (blue). **p** IL-33 expression in clusters 23, 27, and 30 presented as a violin plot grouped by disease status. **q**–**t**, Key surface (**q**–**r**), and lineage-specific (**s**–**t**) genes expressed in IL-33 producing cells. Relative expression levels are indicated on a gradient from low (light gray) to high (blue).

While resistance to infection is likely to involve cytokines such as IFN-γ produced by T cells, many of the symptoms associated with severe COVID-19 appear to be linked to excessive production of innate cytokines such as IL-6 and IL-1β^18^. Consequently, we felt that the results highlighting IL-33, IL-6, IFN-ɑ2, IL-23, and IL-1β production (Fig. 2) could be important. In line with our clustering analysis, we found that all highlighted features were in fact significantly increased in seropositive subjects (Fig. 3a–e). We reasoned that the observed cytokine production (Fig. 3a–d) should be a result of T cell activation, as our experimental strategy favors this mechanism. We found that this could be the case for IL-33, which had a strong positive correlation with CD69 expression in CD4^+^ T cells (Fig. 3f). However, this was not the case for other parameters (Fig. 3g–i) and not unexpectedly for the percentage of IL-6^+^TNF^−^IL-1β^+^ monocytes (Fig. 3j).

To our knowledge, there are no reports associating IL-33 with COVID-19 antibody titers and only one study linking serum IL-33 to increased bone marrow precursors in PBMCs of patients infected with SARS-CoV-2^19^. Given the strong correlation we observed in our data, we decided to explore this result in more detail. We determined that steady-state serum IL-33 was not altered by previous exposure to SARS-CoV-2 in the individuals of our cohort (Supplementary Fig. 4a), nor was the amount of soluble IL-33 receptor ST2 (sST2) changed in sera from seropositive subjects (Supplementary Fig. 4b). As IL-33 interacts with membrane-bound ST2 (ST2L) on immune cells, we next explored which PBMCs were likely to be influenced by the production of this cytokine. Our results indicate that B cells, and to a lesser extent monocytes and dendritic cells, express the most ST2L, both as a percentage of ST2L^+^ cells (Fig. 3k) and by MFI (Fig. 3l–m and Supplementary Fig. 4c), making them potential candidates for the regulation exerted by this cytokine. Finally, we retrieved single-cell RNA sequencing data from a recent publication^20^ and processed it to interrogate *IL33* expression in the setting of SARS-CoV-2 pulmonary disease (Fig. 3n–t and Supplementary Fig. 4d). These data were generated from cells in BALF of 3 healthy donors and 9 acutely SARS-CoV-2 infected patients, 3 of whom displayed mild symptoms, while the remaining 6 were classed as severely ill (Supplementary Fig. 4d). In these data, we identified IL-33 production by 3 distinct cell clusters (Fig. 3n, o). Strikingly, *IL33* expression was confined to SARS-CoV-2 infected patients (Fig. 3p), concomitant with the increased appearance of the *IL33*^+^ cell clusters as a result of disease (Supplementary Fig. 4c). *IL33*^+^ cell clusters were most likely stromal cells (Fig. 3q–t) and are unlikely to be responsible for the IL-33 production we observed in PBMC cultures. For this reason, we investigated the source of IL-33 in PBMCs by staining for intracellular IL-33 in conjunction with cell-specific markers, and found that it was most highly expressed in CD14^+^ monocytes (Supplementary Fig. 5b). However, we observed no differences in the frequency of IL-33^+^ cells in PBMC from seropositive vs. seronegative individuals, suggesting that the emergence of COVID-19-specific T cells that are capable of eliciting the release of bioactive IL-33 from other cells that constitutively make this cytokine may be critical. Nevertheless, these clinical data further implicate IL-33 in COVID-19 immunobiology.

Here, we show that after the resolution of infection, an innate immune cytokine signature persists in PBMCs of convalescent subjects, characterized by induction of IL-33 in response to T cell activation with 14-mer peptides, a peptide length suggestive of CD4^+^ T cell activation^21^. However, the mechanism of activation of the IL-33-producing cells in the PBMCs from convalescent individuals remains unclear at this time. IL-33 was initially described as a cytokine produced as an alarmin by epithelial cells to induce type 2 immune responses^22^, and of interest in this regard, there are reports of eosinophilia, a mark of type 2 immunity, in COVID-19 patients^23^. IL-33 is also linked to lung injury, pulmonary viral infections, and chronic lung diseases^24^. COVID-19 can cause complications such as pneumonia and acute respiratory distress syndrome, as well as invoke lasting damage to the lungs, including lung remodeling and fibrosis, which are consistent with known roles of IL-33^25^. These observations suggest that IL-33 could participate in the pathogenesis of COVID-19, as has recently been discussed in further detail^26^. On the other hand, IL-33 has also been reported to promote antiviral cytotoxic T cell responses^27–29^ and higher antibody production^30,31^. Thus the presence of a persistent population of cells capable of making IL-33 in response to T cell activation may confer an advantage in the case of secondary exposure. Further work on IL-33 will be needed to elucidate the role of this cytokine in COVID-19.

## Methods

### Human subjects

All procedures involving human subjects were approved by the Ethics Committee of the Medical Center–University of Freiburg (305/20). The study was registered at the German Clinical Trial Register (DRKS00022292). Pre-exposure donor serum samples (*n* = 16) had been collected, aliquoted, and stored at the Medical Center-University of Freiburg prior to June 1st, 2019, with written informed consent for biobank storage obtained from each individual. These individuals were considered to be pre-exposure controls since their biological material had been stored prior to the emergence of SARS-CoV-2. This cohort included 50% males (*n* = 8) and 50% females. The median age was 51 years with a range of 39–77 years.

SARS-CoV-2-exposed subjects (*n* = 155) were recruited with a standardized procedure. Individuals who had had a PCR-diagnosed SARS-CoV-2 infection were invited to a COVID-19 aftercare examination by the Occupational Medical Service. Within this examination, individuals were informed about the possibility to participate in the current trial and to also enroll their household members and close contacts who had not been tested for SARS-CoV-2 by PCR or had had a negative PCR result. Inclusion criteria were: contact to SARS-CoV-2-infected individuals, age of at least 5 years, and the ability to provide written informed consent. The exclusion criterium was the presence of symptoms of an acute SARS-CoV-2 infection (fever, cough, malaise) within the last 14 days prior to blood collection. Study participants gave their written informed consent prior to study enrollment. For subjects <18 years of age, written informed consent was provided by one parent. Collection of biological material was performed at the Medical Center–University of Freiburg. Serum and peripheral blood mononuclear cells (PBMCs) were collected at one or two time points. All samples were de-identified and analyzed in a pseudonymized manner. Information on demographic characteristics and disease course were provided by the subjects using a standardized questionnaire filled at the time point of enrollment. Information on age, sex, PCR testing for SARS-CoV-2, symptoms, symptom duration, treatment, and exposure were analyzed. All individuals (*n* = 155) were included in the ‘IgG serology cohort’ (Supplementary Table 1), and for these subjects, anti-SARS-CoV-2-Spike and RBD IgG antibody measurements were performed. From this cohort, *n* = 122 individuals were included in the ‘IgM serology cohort’ (Supplementary Table 1), for which anti-SARS-CoV-2-Spike and RBD IgM antibody measurements were performed. According to the results of the antibody measurement, we selected *n* = 20 individuals who were seronegative (seronegative cohort) and *n* = 20 individuals who were seropositive (‘seropositive cohort) for further analysis of PBMCs (Supplementary Table 2). The demographic and clinical characteristics of all cohorts are provided in Supplementary Tables 1 and 2.

### Serum asservation and ELISA

Serum was collected in tubes containing clot activators (7.5 ml) and stored at room temperature prior to processing. Tubes were centrifuged at 2500 × *g* for 10 min and serum was aliquoted and stored at −20 °C.

### Serology

Collected serum was analyzed for the presence of antibodies against SARS-CoV-2 proteins by adapting a serology assay developed in the laboratory of Dr. Florian Krammer^12^, with some alterations to the protocol. Recombinant spike protein and its receptor-binding domain (RBD) were expressed from mammalian expression plasmids kindly donated by Dr. Krammer^12^. Recombinant proteins were produced employing the Expi293 Expression System (Thermo) following the manufacturer’s recommendations. Briefly, cells were grown to exponential phase in serum-free optimized media, then transfected with plasmid-lipid complexes prepared with Expifectamine. Twenty hours later media were supplemented with expression enhancers and finally, media containing recombinant proteins were harvested 72 h later. Proteins were purified by passing media through Ni-NTA Sepharose columns. The purity and identity of recombinant proteins were established by polyacrylamide gel electrophoresis and mass spectrometry.

96-well flat-bottom plates (Corning) were coated with 2 µg/ml of recombinant proteins overnight at 4 °C. Blocking was performed with 200 µl/well PBS containing 0.05% Tween-20 (PBS/T) and 3% milk for 4 h. Serum samples were diluted 1:50, 1:200, 1:800, and 1:3200 performing serial dilutions in PBS/T containing 1% milk. Serum samples were incubated for 2 h at room temperature. Plates were washed four times with PBS/T and incubated with an anti-human IgG or IgM secondary antibody (dilution 1:3000, Sigma or Thermo Fisher Scientific) for 1 h at room temperature. Plates were washed four times with PBS/T and developed using a TMB substrate kit (Biolegend) for 5 min at room temperature. The colorimetric reaction was stopped by the addition of 2N H_2_SO_4_. Internal positive and negative standards were included on each plate to allow for data normalization between different plates. Absorbance at 450 nm was measured using a TriStar microplate reader (Berthold Technologies).

### PBMC collection

Peripheral blood from 20 seronegative and 20 seropositive subjects (based on anti-Spike IgG ELISA) was collected in EDTA-coated tubes and stored at 4 °C prior to processing. Blood was diluted 1:1 with PBS containing 2% FCS and 1 mM EDTA. Peripheral blood mononuclear cells (PBMCs) were isolated using a density gradient method. Lymphoprep solution (Stem Cell) was pipetted to the bottom of SepMate-50 tubes (Stem Cell), and diluted blood carefully layered on top. After 10 min centrifugation at 1200 × *g*, the upper phase containing mononuclear cells was transferred to a new tube. PBMCs were washed twice with PBS with 2% FCS and 1 mM EDTA. After counting, cells were aliquoted, frozen in FCS containing 10% DMSO, and stored at −80 °C.

### SARS-CoV-2 peptide stimulation

PBMCs were thawed and rested in RPMI containing 10% FCS, 100 U/ml penicillin/streptomycin, 4 mM glutamine, and 55 µM 2-mercaptoethanol for 2 h. 4 × 10^5^ PBMCs were incubated with 2 µg/ml SARS-CoV-2 spike PepMix (JPT) or DMSO control in the presence of 2 µg/ml anti-CD28 and 50 U/ml hIL-2. The PepMix includes a total of 315 14-mer peptides covering the whole spike protein. Incubation was performed for 24 h with the addition of 5 µg/ml Brefeldin A (Biolegend) for the last 4 h to block cytokine secretion. DMSO controls served as negative controls.

### TLR7 agonist stimulation

2 × 10^5^ PBMCs were cultured in RPMI containing 10% FCS, 100 U/ml penicillin/streptomycin, and 4 mM glutamine supplemented with 5 µg/ml Brefeldin A and 5 µg/ml Resiquimod (Invivogen) or water control for 4 h.

### Flow cytometry

Prior to cell surface staining, PBMCs were incubated with purified NA/LE Human BD Fc Block (BD, dilution 1:300) and Live/Dead Fixable Aqua or Live/Dead Fixable Blue (both Thermo Fisher, dilution 1:1000) in FACS buffer (2% FCS and 1 mM EDTA in PBS) for 20 min at 4 °C. Cells were then washed and incubated with cell surface antibody cocktails (dilution 1:200) for 30 min at 4 °C. Cells were washed with FACS buffer and fixed using BD Cytofix Fixation Buffer (for intracellular cytokine staining) or eBioscience Fixation/Permeabilization kit (for Foxp3 staining) for 20 min at room temperature. For intracellular staining assays, cells were washed twice with 1× BD Perm/Wash buffer or 1× eBioscience Perm/Wash buffer prior to incubation with intracellular antibody cocktails (dilution 1:200, except for IL-33 antibodies−1:500) for 45 min at room temperature. Cells were washed twice with 1× BD Perm/Wash buffer or 1× eBioscience Perm/Wash buffer and resuspended in FACS buffer. Flow cytometry data were acquired on a BD LSRFortessa or BD FACSymphony flow cytometer.

### IL-33 intracellular flow cytometry staining

Intracellular IL-33 staining was performed on 250 000 PBMC/well from two seropositive and two seronegative individuals from the cohort. Cells were stained with cell-surface antibodies and fixed/permeabilized using the BD Cytofix/Cytoperm kit as described above. The recombinant anti-IL-33 antibody (clone 002, PE-conjugated, Abcam) was titrated and used at a final concentration of 0.02 µg/well (dilution 1:500). A recombinant Rabbit IgG monoclonal antibody (clone EPR25A, PE-conjugated, Abcam) was used at the same concentration as an isotype control. The staining was performed for 1 h at room temperature. As a second approach, the IL-33 monoclonal antibody (clone 6H617, unconjugated, Invitrogen) was titrated and used at a final concentration of 0.2 µg/well (dilution 1:500). The staining was performed for 1 h at room temperature, followed by incubation with a secondary PE-conjugated rat-anti-mouse antibody (clone A85-1, PE-conjugated, BD Pharmingen, 0.04 µg/well) for 30 min. Control staining was performed by incubating only with the secondary PE-conjugated antibody.

### Cytokine measurement

Supernatants from PBMCs cultured with SARS-CoV-2 PepMix or DMSO control as described above were collected and stored at −20 °C. Cytokines in culture media were detected using the Legendplex™ Human Inflammation Panel 1 (Biolegend, Cat# 740809) following the manufacturer’s instructions. This kit allows for simultaneous measurement of IL-1β, IFNα2, IFNγ, TNF, MCP-1, IL-6, IL-8, IL-10, IL-12p70, IL-17A, IL-18, IL-23, and IL-33. Briefly, standards and cell culture supernatants were incubated on a 96-well V-bottom plate with capture beads for 2 h. The plate was washed and biotinylated detection antibodies were added to each well for 1 h, followed by Streptavidin-phycoerythrin (PE) addition for 30 min. Samples were washed and resuspended in FACS buffer. All steps were carried out at room temperature. Results were obtained using a BD LSRFortessa and a BD FACSymphony A5 flow cytometers and FACSDiva (v. 9.0). Data analysis was performed in FlowJo (v. 10.7). Cytokines were identified based on the size and internal dye of the beads. Cytokine concentration in samples was derived from the geometric mean fluorescence intensity for PE interpolated from standard curves calculated from 8 standard controls measured in duplicate.

IL-33 and sST2 were measured in the serum of subjects from our cohort with commercial kits following the manufacturer’s instructions (Sigma and ThermoFisher, respectively). Briefly, for IL-33 measurement, standards and samples were incubated for 2.5 h at room temperature in pre-coated plates. Plates were then washed 4 times and biotinylated detection antibodies were added for 1 h. Following incubation, plates were washed and Streptavidin-HRP was added to each well for 45 min. For sST2 measurement, standards and samples were incubated for 2 h at room temperature in pre-coated plates with direct addition of biotin-conjugated detection antibodies. Following incubation, plates were washed 4 times with a provided buffer, and Streptavidin-HRP was added to each well. Plates were then incubated for 1 h at room temperature and washed again 4 times. Finally, for both IL-33 and sST2, bound protein was revealed by adding TMB Substrate to each well. Reactions were stopped after 20 min with provided Stop Solution and absorbance read at 450 nm immediately.

### Data analysis and statistics

Clinical data, ELISA readings, flow cytometry results, and single-cell RNA sequencing data were processed and analyzed using R (Lucent Technologies), which was also employed to generate graphs. R packages used included: patchwork (v.1.1.0), reshape2 (v1.4.4), dplyr (v. 0.8.5), CytoExploreR (1.0.8), pheatmap (1.0.12) and ggplot2 (v. 3.3.2).

#### Antibody titers

Background measurements were subtracted from absorbance readings, which were then batch corrected based on internal standards included on each plate. Batch and background corrected data were then used to calculate the area under the dilution curve (AUC) for each subject. To calculate a seropositivity threshold, a subset of AUC values was extracted, composed of all pre-pandemic sera, as well as those subjects with an AUC lower than the maximum obtained from pre-exposure sera and with no SARS-CoV-2 PCR or with a negative PCR result. Positive cases were defined as being at least 2 standard deviations higher than the mean AUC from this sample subset. All other values were defined as seronegative.

#### Symptoms, age, and titer associations

The relationship between antibody titers, subject age, and symptom duration in days was correlated with one another using Kendall rank correlation and reporting $\tau$ as the correlation coefficient. Only complete pairs of observations were used in correlations and all data was scaled by subtracting the mean of each feature and dividing by the corresponding standard deviation. Antibody titers (Ab) were also used to fit logistic regression models capable of predicting the presence of reported symptoms (yes = 1, no = 0) (*S*) incorporating the age (*A*) and sex (Sx) of the subjects, as well as the time between positive PCR and Serology (*t*):

1 $$S \sim {\mathrm{Ab}} + A + {\mathrm{Sx}} + t$$

Models that performed significantly better than the null model were examined for the significant associations between antibody titers and symptoms (*p*-value of less than 0.05 in both conditions). The regression coefficient, the regression coefficient divided by its standard error (z) and *p*-value for Ab titer are reported. Only subjects with a positive PCR and serology (*n* = 51 in IgG cohort and *n* = 42 in IgM cohort) were included in this analysis.

#### Immunophenotyping clustering and regression

Flow cytometry data were analyzed using FlowJo version 10.7. Flow cytometry results were collected and stimulation controls were subtracted from values obtained for treatments. A signal intensity threshold of 5000 was used for CD69, TNF, and IFN-g to define populations with high expression. Corrected values were subsequently scaled and clustered based on Euclidean distances to antibody titers and subject age, which were similarly scaled. Heatmaps and clustering were obtained by using pheatmap v.1.0.12. To complement clustering analysis, correlations were explored between antibody titers and flow cytometry results using Kendall rank correlation and reporting $\tau$ as the correlation coefficient. Selected features were correlated with one another using a linear regression model (*y* ~ *x*) and reporting R^2^ as an indication of the quality of the fit.

Flow cytometry analysis of ST2L expression in PBMCs was carried out using CytoExploreR (R package version 1.0.8. https://github.com/DillonHammill/CytoExploreR) for gating and initial processing, then uwot (R package version 0.1.10 https://cran.r-project.org/web/packages/uwot/index.html) for dimensionality reduction.

#### Single-cell RNA sequencing analysis

The transcriptional profiles of ~90,000 single cells in bronchoalveolar lavage fluid from 3 healthy individuals and 9 SARS-CoV-2 infected patients (3 with mild and 6 with severe disease) were retrieved from a publicly available repository (GSE145926). Matrices with raw unfiltered read counts for detected features in barcoded cells were downloaded for each subject and subsequently processed, analyzed, and visualized in R using Seurat v. 3^32^, removing cells with a high percentage of mitochondrial RNA over total RNA (>25%) and with less than 200 detected features, normalizing gene expression data by applying regularized negative binomial regression^33^ and using a Uniform Manifold Approximation and Projection (UMAP)^34^ as a dimensionality reduction approach. Differentially expressed genes within each cluster and across conditions were determined with Seurat as those with a greater than 1.2 fold change and an adjusted *p*-value of less than 0.05.

### Reporting summary

Further information on research design is available in the Nature Research Reporting Summary linked to this article.

## Supplementary information

Supplementary Information

Reporting Summary

## Data Availability

All data reported in this study has been submitted to the publisher and is available for download. Source data are provided with this paper.

## Source data

Source Data

## Code availability

No custom code was used to generate graphs or analyze data.
