# Supplementary material for: IL-33 expression in response to SARS-CoV-2 correlates with seropositivity in COVID-19 convalescent individuals
Source: Nat Commun. 2021 Apr 9;12:2133. doi: 10.1038/s41467-021-22449-w (PMC8035172; doi:10.1038/s41467-021-22449-w)
Supplement: Supplementary file 1 — Supplementary Information [file 41467_2021_22449_MOESM1_ESM.pdf]

Supplementary Information

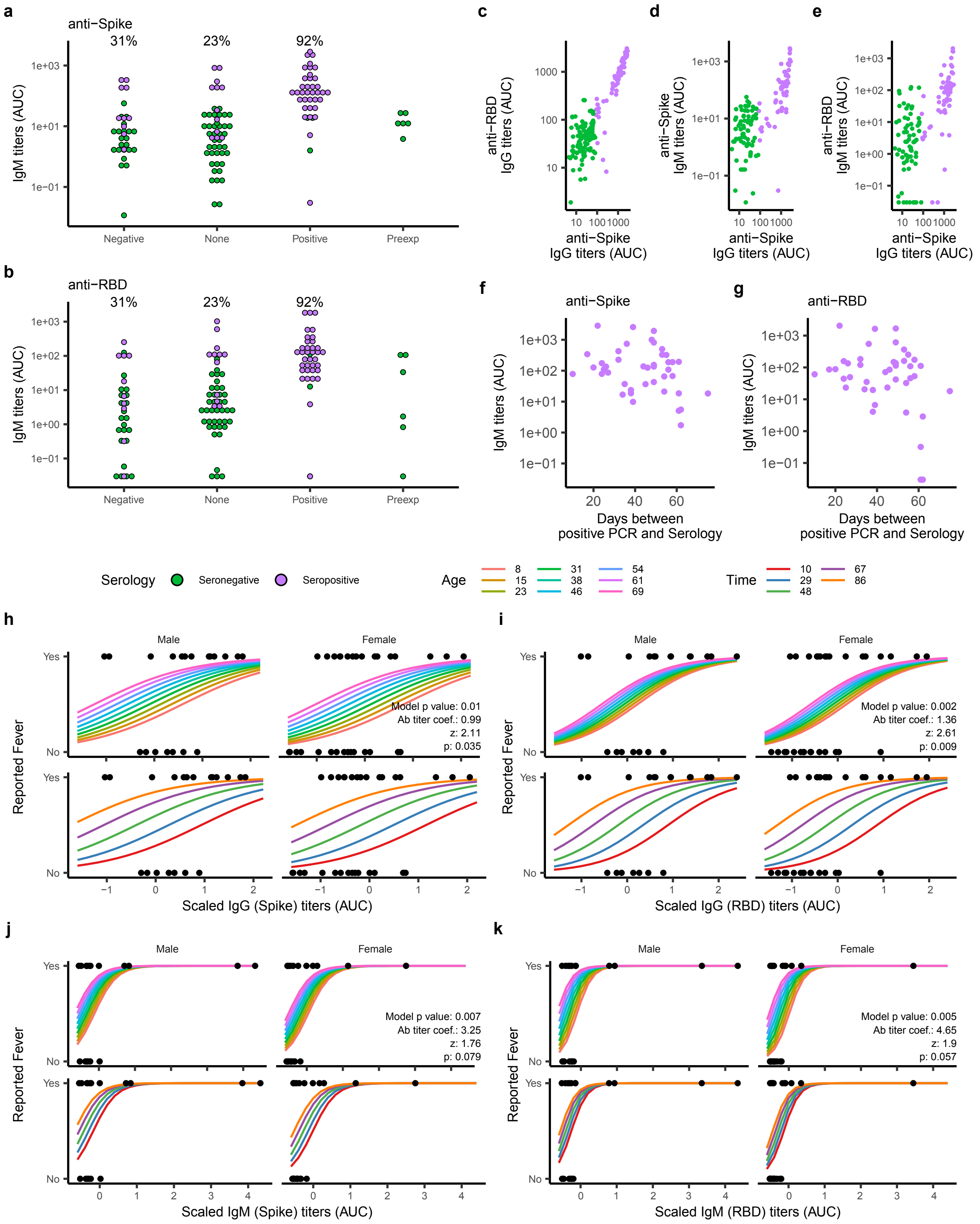

Supplementary Figure 1. Related to Figure 1: Serological characterization of study population.

a–b, Anti-Spike (a) and anti-RBD (b) IgM titers shown as AUC for investigated subjects grouped by SARS-CoV-2 PCR status. Subjects are colored by serology results.

c–e, Indicated antibody titers are shown versus anti-Spike IgG titers, with subjects colored by serology results. Percentage seropositive subjects within each category are indicated.

f–g, Anti-Spike (j) and anti-RBD (k) IgM titers shown as a function of time for seropositive subjects. Approximated time of infection was taken as day of positive SARS-CoV-2 PCR status.

h–k, Detected antibody titers were used to fit logistic regression models to symptom reporting by seropositive subjects, incorporating age, sex and time between positive PCR and serology. Fitted models (lines) are shown for female and male subjects, for different ages with the time variable fixed to population mean (top), or for different times with the age variable fixed to the population mean (bottom). The p value corresponding to the comparison of the model to the null model using a Chi squared test of goodness of fit, as well as the regression coefficient, z and p value (two tailed T-test) associated with the antibody titers are indicated.

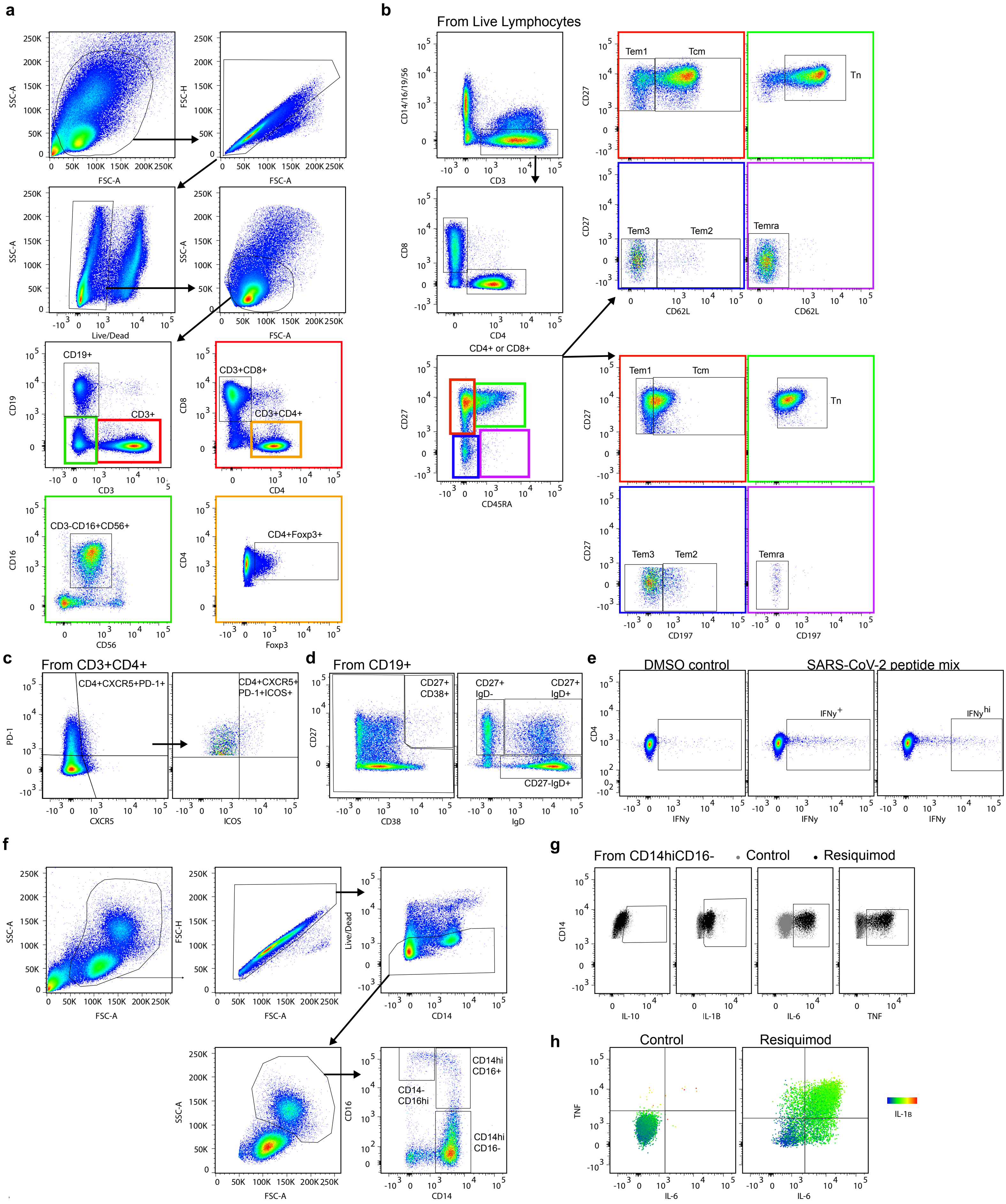

a
b
From Live Lymphocytes
c
From CD3+CD4+
d
From CD19+
e
DMSO control
SARS-CoV-2 peptide mix
f
g
From CD14hiCD16-
Control
Resiquimod
h
Control
Resiquimod
SSC-A
FSC-A
FSC-H
Live/Dead
CD19+
CD3+
CD19
CD3
CD3+CD8+
CD3+CD4+
CD8
CD4
CD3-CD16+CD56+
CD16
CD56
CD4+Foxp3+
Foxp3
CD14/16/19/56
CD4+ or CD8+
CD27
CD45RA
Tem1
Tcm
Tn
Tem3
Tem2
Temra
CD62L
CD197
CD4+CXCR5+PD-1+
CD4+CXCR5+
PD-1+ICOS+
PD-1
CXCR5
ICOS
CD27+
CD38+
CD38
CD27+
IgD-
CD27+
IgD+
CD27-IgD+
IgD
IFNy+
IFNyhi
IFNy
CD14
CD14-
CD16hi
CD14hi
CD16+
CD14hi
CD16-
IL-10
IL-1B
IL-6
TNF
IL-1B

Supplementary Figure 2. Related to Figure 2: PBMCs from convalescent and non-infected subjects differ only in innate cytokine production profile.

a–e, Gating strategies for PBMCs shown as representative flow cytometry dot plots for lymphocytes (a), $CD4^+$ and $CD8^+$ memory cell populations (b), T follicular helper cells (c), B cells (d), lymphocyte cytokine production (e), monocyte populations (f) and monocyte cytokine production (g–h). Control and treatments results are shown for stimulation experiments (e, g–h).

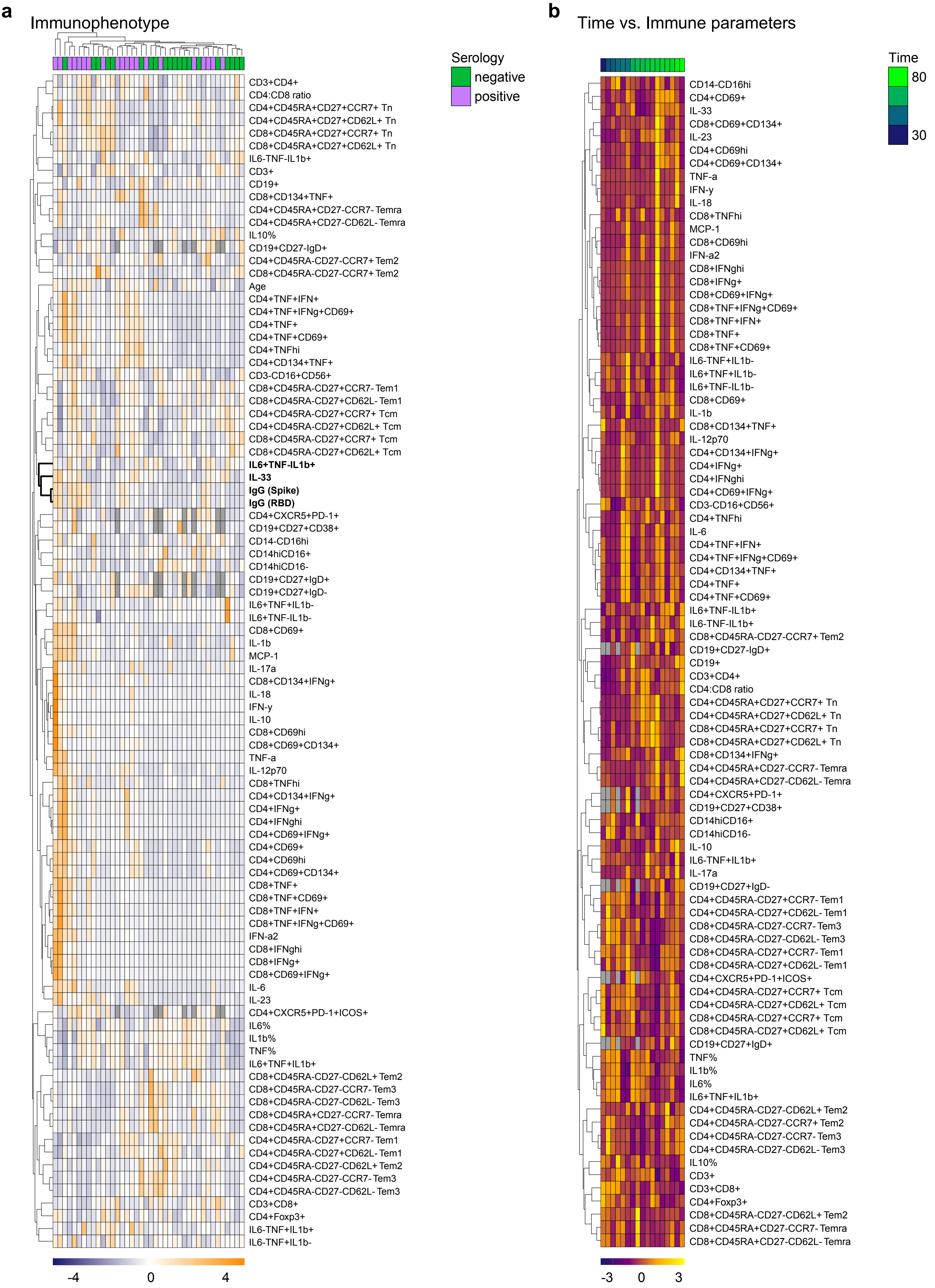

a
Immunophenotype
Serology
negative
positive
CD3+CD4+
CD4:CD8 ratio
CD4+CD45RA+CD27+CCR7+ Tn
CD4+CD45RA+CD27+CD62L+ Tn
CD8+CD45RA+CD27+CCR7+ Tn
CD8+CD45RA+CD27+CD62L+ Tn
IL6-TNF-IL1b+
CD3+
CD19+
CD8+CD134+TNF+
CD4+CD45RA+CD27-CCR7- Temra
CD4+CD45RA+CD27-CD62L- Temra
IL10%
CD19+CD27-IgD+
CD4+CD45RA-CD27-CCR7+ Tem2
CD8+CD45RA-CD27-CCR7+ Tem2
Age
CD4+TNF+IFN+
CD4+TNF+IFNg+CD69+
CD4+TNF+
CD4+TNF+CD69+
CD4+TNFhi
CD4+CD134+TNF+
CD3-CD16+CD56+
CD8+CD45RA-CD27+CCR7- Tem1
CD8+CD45RA-CD27+CD62L- Tem1
CD4+CD45RA-CD27+CCR7+ Tcm
CD4+CD45RA-CD27+CD62L+ Tcm
CD8+CD45RA-CD27+CCR7+ Tcm
CD8+CD45RA-CD27+CD62L+ Tcm
IL6+TNF-IL1b+
IL-33
IgG (Spike)
IgG (RBD)
CD4+CXCR5+PD-1+
CD19+CD27+CD38+
CD14-CD16hi
CD14hiCD16+
CD14hiCD16-
CD19+CD27+IgD+
CD19+CD27+IgD-
IL6+TNF+IL1b-
IL6+TNF-IL1b-
CD8+CD69+
IL-1b
MCP-1
IL-17a
CD8+CD134+IFNg+
IL-18
IFN-y
IL-10
CD8+CD69hi
CD8+CD69+CD134+
TNF-a
IL-12p70
CD8+TNFhi
CD4+CD134+IFNg+
CD4+IFNg+
CD4+IFNghi
CD4+CD69+IFNg+
CD4+CD69+
CD4+CD69hi
CD4+CD69+CD134+
CD8+TNF+
CD8+TNF+CD69+
CD8+TNF+IFN+
CD8+TNF+IFNg+CD69+
IFN-a2
CD8+IFNghi
CD8+IFNg+
CD8+CD69+IFNg+
IL-6
IL-23
CD4+CXCR5+PD-1+ICOS+
IL6%
IL1b%
TNF%
IL6+TNF+IL1b+
CD8+CD45RA-CD27-CD62L+ Tem2
CD8+CD45RA-CD27-CCR7- Tem3
CD8+CD45RA-CD27-CD62L- Tem3
CD8+CD45RA+CD27-CCR7- Temra
CD8+CD45RA+CD27-CD62L- Temra
CD4+CD45RA-CD27+CCR7- Tem1
CD4+CD45RA-CD27+CD62L- Tem1
CD4+CD45RA-CD27-CD62L+ Tem2
CD4+CD45RA-CD27-CCR7- Tem3
CD4+CD45RA-CD27-CD62L- Tem3
CD3+CD8+
CD4+Foxp3+
IL6-TNF+IL1b+
IL6-TNF+IL1b-
-4
0
4
b
Time vs. Immune parameters
Time
80
30
CD14-CD16hi
CD4+CD69+
IL-33
CD8+CD69+CD134+
IL-23
CD4+CD69hi
CD4+CD69+CD134+
TNF-a
IFN-y
IL-18
CD8+TNFhi
MCP-1
CD8+CD69hi
IFN-a2
CD8+IFNghi
CD8+IFNg+
CD8+CD69+IFNg+
CD8+TNF+IFNg+CD69+
CD8+TNF+IFN+
CD8+TNF+
CD8+TNF+CD69+
IL6-TNF+IL1b-
IL6+TNF+IL1b-
IL6+TNF-IL1b-
CD8+CD69+
IL-1b
CD8+CD134+TNF+
IL-12p70
CD4+CD134+IFNg+
CD4+IFNg+
CD4+IFNghi
CD4+CD69+IFNg+
CD3-CD16+CD56+
CD4+TNFhi
IL-6
CD4+TNF+IFN+
CD4+TNF+IFNg+CD69+
CD4+CD134+TNF+
CD4+TNF+
CD4+TNF+CD69+
IL6+TNF-IL1b+
IL6-TNF-IL1b+
CD8+CD45RA-CD27-CCR7+ Tem2
CD19+CD27-IgD+
CD19+
CD3+CD4+
CD4:CD8 ratio
CD4+CD45RA+CD27+CCR7+ Tn
CD4+CD45RA+CD27+CD62L+ Tn
CD8+CD45RA+CD27+CCR7+ Tn
CD8+CD45RA+CD27+CD62L+ Tn
CD8+CD134+IFNg+
CD4+CD45RA+CD27-CCR7- Temra
CD4+CD45RA+CD27-CD62L- Temra
CD4+CXCR5+PD-1+
CD19+CD27+CD38+
CD14hiCD16+
CD14hiCD16-
IL-10
IL6-TNF+IL1b+
IL-17a
CD19+CD27+IgD-
CD4+CD45RA-CD27+CCR7- Tem1
CD4+CD45RA-CD27+CD62L- Tem1
CD4+CD45RA-CD27-CCR7- Tem3
CD8+CD45RA-CD27-CD62L- Tem3
CD8+CD45RA-CD27+CCR7- Tem1
CD8+CD45RA-CD27+CD62L- Tem1
CD4+CXCR5+PD-1+ICOS+
CD4+CD45RA-CD27+CCR7+ Tcm
CD4+CD45RA-CD27+CD62L+ Tcm
CD8+CD45RA-CD27+CCR7+ Tcm
CD8+CD45RA-CD27+CD62L+ Tcm
CD19+CD27+IgD+
TNF%
IL1b%
IL6%
IL6+TNF+IL1b+
CD4+CD45RA-CD27-CD62L+ Tem2
CD4+CD45RA-CD27-CCR7+ Tem2
CD4+CD45RA-CD27-CCR7- Tem3
CD4+CD45RA-CD27-CD62L- Tem3
IL10%
CD3+
CD3+CD8+
CD4+Foxp3+
CD8+CD45RA-CD27-CD62L+ Tem2
CD8+CD45RA+CD27-CCR7- Temra
CD8+CD45RA+CD27-CD62L- Temra
-3
0
3

Supplementary Figure 3. Related to Figure 2: PBMCs from convalescent and non-infected subjects differ only in innate cytokine production profile.

a–b, PBMCs in blood collected from a subset of 20 seropositive and 20 seronegative subjects were isolated, analyzed via flow cytometry for cellular composition and stimulated with a SARS-CoV-2 spike peptide mix or vehicle control, or with TLR7 agonist Resiquimod or vehicle control. Activation of $CD3^{+}CD4^{+}$, $CD3^{+}CD8^{+}$ and $CD14^{hi}CD16^{-}$ cells plus cytokine secretion into culture media were measured via flow cytometry. Control corrected (treatment - control) scaled data ($\frac{x_i - \mu}{\sigma}$) is shown in a single clustered heatmap, where scaled anti-Spike and anti-RBD IgG titers plus subject age are included (a), or where serpositive subjects are sorted based on time between SARS-CoV-2 positive PCR status and PBMC collection (b). Data was clustered based on Euclidean distances between features (rows) or subjects (columns), with clusters indicated with dendrograms. Serology results are included for each subject (seropositive – purple; seronegative – green) and a time scale is provided. Features most closely associated with antibody titers are highlighted in bold letters. Missing values are shown in dark grey.

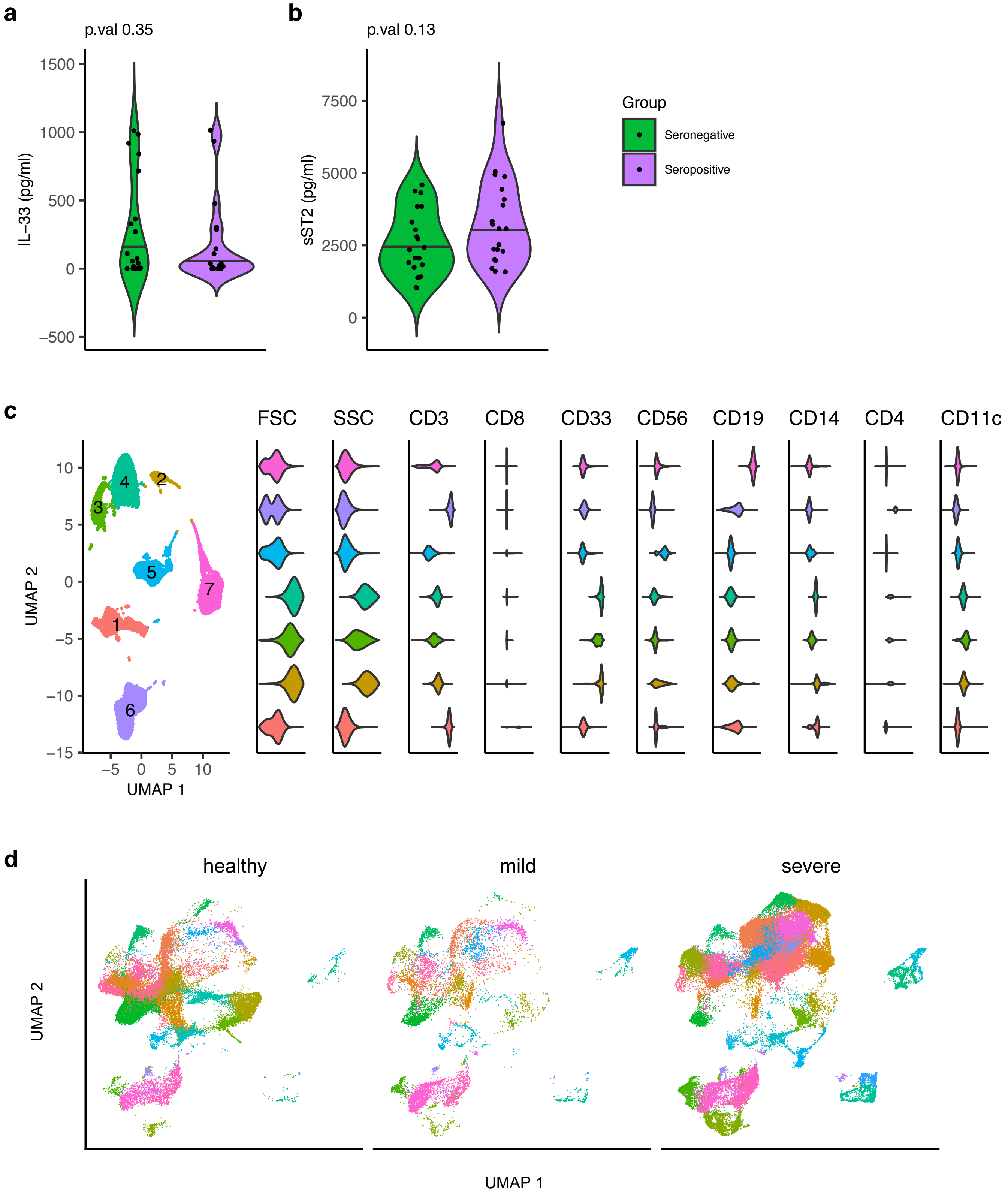

a
p.val 0.35
IL−33 (pg/ml)
b
p.val 0.13
sST2 (pg/ml)
Group
Seronegative
Seropositive
c
FSC
SSC
CD3
CD8
CD33
CD56
CD19
CD14
CD4
CD11c
UMAP 2
UMAP 1
d
healthy
mild
severe
UMAP 2
UMAP 1

Supplementary Figure 4. Related to Figure 3: IL-33 production correlates with T cell activation and disease severity SARS-CoV-2 in infected subjects.

a–b, IL-33 and sST2 were measured in the sera from seropositive (purple) and seronegative (green) subjects. Two tailed Mann-Whitney U tests were calculated and resulting p values are reported. Values for subjects are shown as dots, the sample distribution is presented as a violin plot and the population median as a black line.

c, Flow cytometry staining is presented in a dimensional reduction projection (UMAP), showing cell clusters (left) and relative expression of key markers in each cluster as violin plots (right).

d, Single cell RNA sequencing data from the bronchoalveolar lavage fluid of 3 healthy individual compared to 9 SARS-CoV-2 infected subjects with different disease severities (3 mild; 6 severe) was retrieved from a public database (GSE145926), grouped into cell clusters and shown here by disease status.

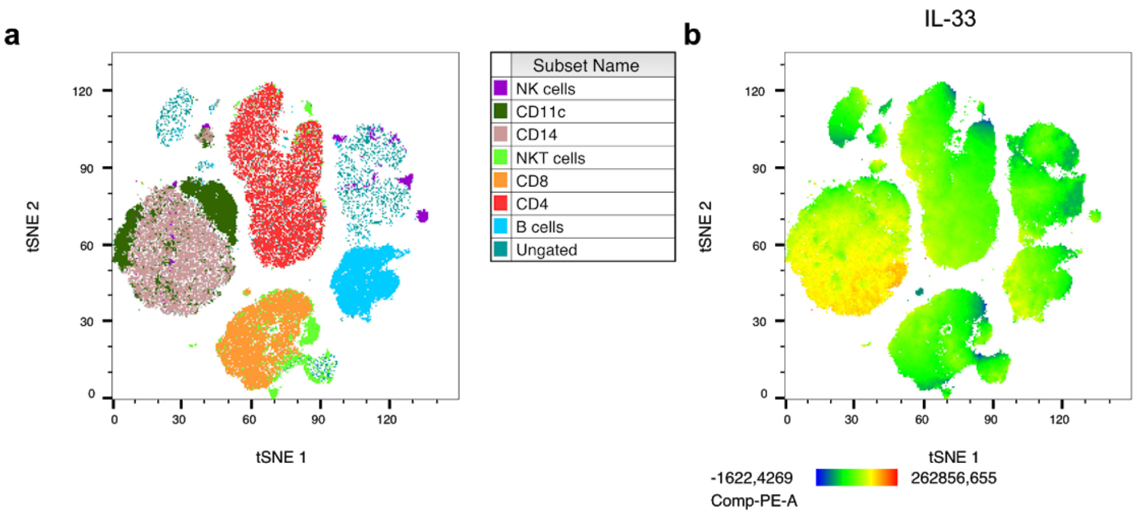

Supplementary Figure 5. Related to Figure 3: IL-33 expression in PBMCs.

a–b, IL-33 was measured in the indicated PBMC populations via flow cytometry. Signal intensities are presented in a t-distributed stochastic neighbouring embedding (tSNE) showing immune clusters (a) and IL-33 expression (b).

Supplementary Table 1. Demographics and disease characteristics serology cohorts

Characteristic	IgG serology cohort	IgM serology cohort
**Demographics**		
No. participants	155	122
Median age (range), years	36 (5–79)	34 (5–62)
Sex<br>female - no. (%)<br>male - no. (%)	 98 (63.2)<br>57 (36.8)	 77 (63.1)<br>45 (36.9)
Age<br>0-17<br>>18	 14 (9)<br>141 (91)	 13 (10.7)<br>109 (89.3)
**Disease characteristics**		
Exposure<br>Professional<br>Household<br>Professional and household<br>Other	 99 (64.5)<br>44 (28.4)<br>7 (3.9)<br>5 (3.2)	 82 (67.2)<br>30 (24.5)<br>5 (4.1)<br>5 (4.1)
Symptoms in the last 16 weeks prior serology - no. (%)	97 (62.6)	74 (60.7)
Median duration of symptoms - days (range)	10 (1–63)	11 (1–55)
SARS-CoV-2 PCR<br>Positive - no. (%)<br>Negative - no. (%)<br>Not performed - no. (%)	 47 (30.3)<br>40 (25.8)<br>68 (43.9)	 38 (31.1)<br>32 (26.2)<br>52 (42.7)
Median time between positive PCR and positive serology - days (range)	49 (10–84)	45.5 (10–61)
**Symptoms**		
Cephalgia<br>Prevalence - no. (%)<br>Median duration - days (range) [missing]	 65 (41.9)<br>6 (1–21)	 50 (41)<br>6 (1–21) [4]

Malaise		
Prevalence - no. (%)	63 (40.6%)	46 (37.7)
Median duration - days (range) [missing]	10 (1–63)	10 (1–48) [2]
Cough		
Prevalence - no. (%)	55 (35.5)	42 (34.4)
Median duration - days (range) [missing]	7 (1–21)	7 (1–20) [6]
Fever		
Prevalence - no. (%)	49 (31.6)	34 (27.9)
Median duration - days (range) [missing]	4 (1–45)	4 (1–14) [5]
Musculoskeletal pain		
Prevalence - no. (%)	43 (27.7)	30 (24.5)
Median duration - days (range) [missing]	7 (1–18)	6.5 (1–18) [3]
Anosmia		
Prevalence - no. (%)	40 (25.8)	29 (23.8)
Median duration - days (range) [missing]	7.5 (2–55)	14 (1–55) [4]
Pharyngitis		
Prevalence - no. (%)	37 (23.9)	31 (25.4)
Median duration - days (range) [missing]	3.5 (1–20)	2.75 (1–14) [4]
Dyspnea		
Prevalence - no. (%)	30 (19.4)	24 (19.7)
Median duration - days (range) [missing]	10 (1–21)	10 (1–21) [10]
Weight loss		
Prevalence - no. (%)	24 (15.5)	17 (13.9)
Median value - kg (range) [missing]	3.5 (1–10)	4.25 (1–10) [4]
**Treatment**		
Hospitalization	3 (1.9)	1 (0.8)
Medication		
Hydroxychloroquine	2 (1.3)	1 (0.8)
Antipyretics	17 (11)	14 (11.5)

Supplementary Table 2. Demographics and disease characteristics PBMC analysis cohorts

Characteristic	Seropositive cohort	Seronegative cohort
**Demographics**		
No. participants	20	20
Median age (range), years	51.5 (14–58)	37.5 (19–75)
Sex<br>female - no. (%)<br>male - no. (%)	 9 (45)<br>11 (55)	 12 (60)<br>8 (40)
**Disease characteristics**		
Median duration of symptoms - days (range)	15.5 (3–55)	0 (0–21)
SARS-CoV2 PCR<br>Positive - no. (%)<br>Negative - no. (%)<br>Not performed - no. (%)	 18 (90)<br>0 (0)<br>2 (10)	 0 (0)<br>8 (40)<br>12 (60)
SARS-CoV-2 anti-Spike IgG<br>Positive - no. (%)<br>Negative - no. (%)	 20 (100)<br>0 (0)	 0 (0)<br>20 (100)
Median time between positive PCR and PBMC collection - days (range)	66 (29–85)	n.a.

Supplementary Table 3. Key reagents

Reagent	Company	Identifier
**Antibodies for ELISA**		
Goat anti-Human IgG (H+L) Secondary Antibody, HRP	ThermoFisher Scientific	Cat# 31410
Anti-Human IgM (μ-chain specific)−Peroxidase antibody produced in goat	Sigma	Cat# A0420-1ml
**Materials for PBMC isolation**		
Lymphoprep	Stem Cell	Cat# 07851
SepMate 50 ml tube	Stem Cell	Cat# 85450
**Virus peptide mixes**		
PepMix SARS-CoV-2 Spike Glycoprotein	JPT	PM-WCPV-S-1
PepMix HCMVA (pp65)	JPT	PM-PP65-2
**Antibodies for flow cytometry**		
AlexaFluor647 anti-human CD3	Biolegend	Clone: HIT3a<br>Cat# 300322
PE anti-human CD3	Biolegend	Clone: HIT3a<br>Cat# 300308
BrilliantViolet 711 anti-human CD4	Biolegend	Clone: OKT4<br>Cat# 317440
FITC anti-human CD4	Biolegend	Clone: A161A1<br>Cat# 357406
Pacific Blue anti-human CD8	Biolegend	Clone: HIT8a<br>Cat# 300928
APC anti-human CD8	Biolegend	Clone: HIT8a

		Cat# 300812
BrilliantViolet 510 anti-human CD14	Biolegend	Clone: M5E2<br>Cat# 301842
APC-Cy7 anti-human CD14	Biolegend	Clone: M5E2<br>Cat# 301820
BrilliantViolet 605 anti-human CD16	Biolegend	Clone: 3G8<br>Cat# 302039
APC-Cy7 anti-human CD16	Biolegend	Clone: 3G8<br>Cat# 302017
PE anti-human CD19	Biolegend	Clone: HIB19<br>Cat# 302208
APC-Cy7 anti-human CD19	Biolegend	Clone: HIB19<br>Cat# 302217
PE/Dazzle 594 anti-human CD27	Biolegend	Clone: M-T271<br>Cat# 356421
PE-Cy7 anti-human CD45RA	Biolegend	Clone: HI100<br>Cat# 304112
APC-Cy7 anti-human CD56	Biolegend	Clone: HCD56<br>Cat# 318332
AlexaFluor 488 anti-human CD62L	Biolegend	Clone: DREG56<br>Cat# 304816
BrilliantViolet 785 anti-human CD69	Biolegend	Clone: FN50<br>Cat# 310932
PE/Dazzle 594 anti-human CD134	Biolegend	Clone: ACT35<br>Cat# 350019
BrilliantViolet 785 anti-human CD197	Biolegend	Clone: G043H7<br>Cat# 353230
AlexaFluor 488 anti-human FOXP3	Biolegend	Clone: 206D<br>Cat# 320111
AlexaFluor647 anti-human IFNg	Biolegend	Clone: 4SB3<br>Cat# 502516
AlexaFluor700 anti-human TNF	Biolegend	Clone: MAb11<br>Cat# 502928
PE-Cy7 anti-human TNF	eBioscience	Clone: TN3-19.12<br>Cat# 25-7423-82

FITC anti-human IL-1 beta	eBioscience	Clone: CRM56<br>Cat# 11-7018-42
eFluor 450 anti-human IL-6	eBioscience	Clone: MQ2-13A5<br>Cat# 48-7069-42
APC anti-human IL-10	Biolegend	Clone: JES3-9D7<br>Cat# 501409
Alexa Fluor 488 anti-human CD11c	Biolegend	Clone: 3.9<br>Cat# 301618
Alexa Fluor 647 anti-human CD185 (CXCR5)	Biolegend	Clone: J252D4<br>Cat# 356906
PE-Cy7 anti-human IgD	Biolegend	Clone: IA6-2<br>Cat# 348210
PE-Cy7 anti-human CD33	Biolegend	Clone: P67.6<br>Cat# 366617
Brilliant Violet 785 anti-human CD278 (ICOS)	Biolegend	Clone: C398.4A<br>Cat# 313533
PE anti-human PD-1	Biolegend	Clone: EH12.2H7<br>Cat# 329905
FITC anti-human ST2L	mdbioscience	Clone: B4E6<br>Cat# 101002F
Brilliant Violet 650 anti-human CD38 antibody	Biolegend	Clone: HB-7<br>Cat# 356619
PE recombinant anti-IL-33 antibody	Abcam	Clone: 002<br>Cat# ab275607
PE recombinant Rabbit IgG	Abcam	Clone: EPR25A<br>Cat# ab209478
IL-33 monoclonal antibody	Invitrogen	Clone: 6H617<br>Cat# MA5-16242
PE rat anti-mouse IgG1	BD Pharmingen	Clone: A85-1<br>Cat# 550083
**Other reagents for flow cytometry**		
Live/Dead Fixable Aqua Dead Cell Stain Kit	ThermoFisher Scientific	Cat# 34957

Live/Dead Fixable Blue Dead Cell Stain Kit	ThermoFisher Scientific	Cat# L34962
Purified NA/LE Human BD Fc block	BD	Cat# 564765
**Commercial kits**		
TMB substrate set	Biolegend	Cat# 421101
Foxp3/Transcription factor staining buffer set	eBioscience	Cat# 00-5523-00
Fixation/Permeabilization Solution Kit	BD	Cat# 554714
LEGENDplex Human Inflammation Panel 1	Biolegend	Cat# 740809
ST2 (IL-33R) Human ELISA Kit	ThermoFisher	Cat# BMS2231
Human IL-33 ELISA Kit	Sigma	Cat# RAB0297-1KT

Supplementary Note 1. Study protocol 'Serological screening and metabolic analysis for evaluation of the immune response to SARS-CoV2'

1. Project title and version

Project title:	Serological screening and metabolic analysis for evaluation of the immune response to SARS-CoV2
Version:	3.0

2. Summary

SARS-CoV2 is a single stand RNA virus that belongs to the coronavirus family. This virus is infectious for humans and can lead to a disease of the upper and lower respiratory tract, known as COVID-19. Symptoms of this disease are fever, pharyngitis, dyspnea. In severe cases, a fulminant, life-threatening pneumonia can develop. Due to its rapid spread, SARS-CoV-2 poses an unprecedented challenge for the health care system, economy and society in many countries. So far there is only limited data what percentage of the population have already had contact with the virus and have developed an immune response. There is insufficient knowledge why some people experience severe symptoms whereas others have only mild symptoms and still other infections are completely asymptomatic. In this project, we aim to develop an ELISA-based antibody test, which allows a quantification of the antibody titer. With this test, we are going to analyze our study population and test which participants have antibodies against SARS-CoV2. We are going to enroll individuals who have had an exposure to SARS-CoV-2 either in a professional setting or in their household, regardless of whether these individuals have had or not had a positive SARS-CoV2 PCR test. With this study we can assess if there have been asymptomatic infections. We also aim to include study subjects who live in the same household as participants with a PCR-proven SARS-CoV2 infection. This analysis would allow us to describe the infection rate in households of infected individuals. We aim to perform correlations of the antibody titers with the clinical manifestation of disease. After a first screening test, we are going to select individuals for further scientific analyses. We are going to focus on examining the virus-specific T cells as well as metabolic characteristics of immune cells. We aim to analyze if there is a correlation between metabolic features, immune cell function and clinical course of COVID-19.

3. **Investigators**

Principal Investigator	Prof. Dr. C. Waller<br>Department of Hematology, Oncology and Stem Cell transplantation<br>Medical Center - University of Freiburg<br>Hugstetter Str. 55<br>79106 Freiburg
Sub-Investigators	Dr. Petya Apostolova<br>Department of Hematology, Oncology and Stem Cell transplantation<br>Medical Center - University of Freiburg<br>Hugstetter Str. 55<br>79106 Freiburg<br><br>Dr. Daniel Steinmann<br>Occupational Medicine<br>Medical Center - University of Freiburg<br>Breisacher Str. 86b<br>79110 Freiburg<br><br>Dr. Erika Pearce<br>Max-Planck Institute for Immunobiology and Epigenetics<br>Stübeweg 51<br>79108 Freiburg
Institutions	Medical Center - University of Freiburg<br>Department of Hematology, Oncology and Stem Cell transplantation<br>Hugstetter Str. 55<br>79106 Freiburg<br><br>Medical Center - University of Freiburg<br>Occupational Medicine<br>Breisacher Str. 86b<br>79110 Freiburg<br><br>Max-Planck Institut for Immunobiology and Epigenetics

	Stübeweg 51<br>79108 Freiburg

The study is financed with intramural funding. The study has not been registered in a public registry so far. (note: registration upon receipt of IRB approval)

4. Scientific background

SARS-CoV2 is a single stand RNA virus that belongs to the coronavirus family. This virus can replicate in the mucosa of the nose and throat and can infect the alveolar epithelial cells of the lung. In some individuals, an infection with SARS-CoV2 has a mild course; other patients develop a severe pneumonia and require mechanical ventilation.

The diagnostic procedure for a SARS-CoV2 infection is a PCR from a nose-throat swab. The infection is often diagnosed only in individuals with severe symptoms, as individuals with mild or no symptoms do not always get tested. For this reason, it is unclear what percentage of the population and especially in the households of SARS-CoV2 infected people, has actually been in contact with the virus. After an infection, IgM and IgG antibodies develop. They can be measured by a serology test (ELISA). Such tests are not part of the routine diagnostic procedure yet. It is not clear whether the antibody titers have a correlation with the symptoms and how the antibody titers change over time.

A successful immune response against SARS-CoV2 also requires an intact T cell immunity. During their immune response against a virus, T cells undergo different stages of development. Naive T cells become effector T cells and after the infection is successfully cleared they can build a memory cell population. These different phases are tightly connected to changes in the T cell metabolism. Naive T cells employ mostly oxidative phosphorylation to cover their energetic needs. During the effector phase, glycolysis is activated. Memory T cells use mostly fatty acid oxidation and oxidative phosphorylation. This switch between different metabolic processes is essential for proper T cell function.

In this project, we aim to develop an ELISA test for antibody screening. Subjects professionally exposed to SARS-CoV2 will get informed about the possibility to participate in this trial by their Occupational Medicine physician. Other individuals will be recruited as described in section 7. We are going to conduct an IgM and IgG ELISA for antibodies against the spike protein or the receptor-binding domain (RBD) of the spike protein. For individuals with a positive antibody test, we would offer the serology test to other persons living in the same household. Furthermore, we are going to investigate whether SARS-CoV2 specific T cells are present in convalescent individuals and are going to assess the metabolism of these T cells.

We aim to answer following questions:

1. What percentage of the study population has been in contact with SARS-CoV2? Are antibodies present in individuals that did not have a PCR-proven infection?
2. What is the transmission rate in the households of SARS-CoV2 infected indivduals?
3. Is there a correlation between antibody titer and symptoms?
4. Are virus-specific memory T cells present in convalescent individuals?
5. Is there a correlation between metabolic parameters in serum and immune cells and symptoms?

5. Aims of the project

The primary aim of the project is to develop an ELISA-based serology test that allows to quantify the antibody titer. With this test, we are going to study the rate of seropositivity in our study population. Furthermore, we are going to investigate whether there is a correlation between the antibody titer and the symptom severity and duration. Another aim of the project is to quantify the virus-specific T cells in convalescent individuals and to study their metabolism. We are going to investigate a possible correlation between immune cell metabolism and symptoms. The long-term goal of this project is to gain new insights into the immune response to SARS-CoV2.

6. Outcomes

Primary outcomes are the anti-SARS-CoV2 IgG and IgM antibody titers (quantified as area under the curve, AUC from several dilution of a serum sample). For some of the experiments we aim to quantify the amount of virus-specific T cells as well as metabolites in immune cells and serum of the study subjects using liquid chromatography-mass spectrometry.
Secondary outcomes are the rate of seropositivity in the study population, the correlation between antibody titer and virus-specific T cells and symptoms as well as the correlation between metabolic parameters, antibody titers and symptoms.

7. Study population

The maximal number of recruited subjects will be 250.
In a first step, we are going to include up to 25 participants whose serum has been stored in the biobanks HBUF or FREEZE prior to 01.06.2019. These samples will serve as a historic control for the validation of the antibody test. As the samples have been collected prior to the

emergence of SARS-CoV2, these samples should have a negative result in the antibody test. We are going to use 1 ml serum or plasma.
Furthermore, we are going to include at least 25 subjects who had a positive SARS-CoV2 PCR test and whose blood has been stored in the biobanks HBUF or FREEZE. These samples will serve as positive controls. The technical quantification of the antibody titer and the validation are described in section 10 (biometry).

The next subject group will include individuals that have been exposed to SARS-CoV2 in a professional setting. These individuals will be contacted per e-mail from a physician from the Occupational Medicine Department. Using a standardized e-mail text, we are going to inform the individuals about the possibility to participate in our study. These individuals can then contact the principal investigator. A maximum of 50 ml blood will be collected.
Other individuals will be recruited as household members of persons who have been infected with SARS-CoV2. When providing the information about the trial, we will inform potential study subjects that their household members can be tested as well, should they be interested. The household members will then contact the principal investigators to receive information about the trial. For these individuals, a maximum of 50 ml blood will be collected (exception: minors, see below).
Depending on the result of the scientific tests, the blood collection can be repeated three times within one year.
Our study population includes children and adolescents between 5 and 17 years of age. Including this subpopulation is important in order to assess the rate of infection within a single household. So far there is only limited data about infection transmission within households. This information is of high relevance for epidemiology and society. With this project, we aim to describe the transmission rate within households including children and adolescents.
For individuals younger than 18 years, we will collect 5 ml blood. We are going to perform an antibody screening test for antibodies against SARS-CoV2. In the case of a positive antibody test, we would like to collect blood at two more time points within a year, given the consent of the study subject and the parents.

Inclusion criteria:

- age at leat 5 years
- signed written informed consent (for minors signed by a parent or legal guardian)

Exclusion criteria:

- acute symptoms of an active COVID-19 infection (fever, cough, dyspnea) within the last two weeks prior to enrollment.

8. Methods and study plan

This is a monocentric observational exploratory study.

Participants will receive information about the trial by the principal investigator or an authorized physician using the written inform consent form. All participants will provide their oral and written consent.

All data will be recorded in a pseudonymized manner. Each individual will be assigned a pseudonym (Subject-ID).

After providing the written informed consent, participants will answer several questions regarding infection symptoms, treatment, comorbidities using a standardized questionnaire. Up to 50 ml blood (in EDTA-coated or serum tubes) will be collected (exception: for minors 5 ml). Peripheral blood mononuclear cells (PBMC) will be extracted and frozen or used immediately for experiments. Serum will be isolated and frozen or immediately used for experiments. One part of the experiments will be performed at the Max-Planck Institute for Immunobiology and epigenetics. Up to 7.5 ml serum will be provided for the conduction of the ELISA test. For tests on PBMC including liquid chromatography-mass spectrometry, up to $5x10^6$ PBMC will be provided.

Our aim is to include up to 250 subjects per year and complete the study within 1 year from initiation (i.e. second quarter of 2021).

9. Risk and benefit analysis

The participants have the opportunity to learn, whether they have been in contact with SARS-CoV2 and have developed antibodies. Beyond that, this study might lead to new insights into the immune response to SARS-CoV2.

The study participants are exposed to low risks during the collection of blood. This might include pain or hemorrhage. Thrombosis (blood clots in the vessels), damage of arteries, other blood vessels or nerves and infections are very rare but cannot be completely excluded. Depending on the amount of blood collected, participants can experience a reversible nausea and lack of energy.

The risk-benefit analysis of the study is positive. New advances in the understanding of anti-SARS-CoV2 immunity can be achieved at the cost of low risks. These data might contribute to improving the prevention and treatment strategies against SARS-CoV2.

10. Statistics/Biometry

This is an observational explorative study to characterize the antibody response to SARS-CoV2 within the study population. Using a research laboratory-developed ELISA test, we will quantify IgM, IgG and IgA antibodies against SARS-CoV2. We will use individuals with a positive SARS-CoV2 PCR (n=25) whose blood samples have been stored in the HBUF or FREEZE biobank. We are going to use serum samples collected prior to 01.06.2019 in the HBUF or FREEZE biobank as negative controls. These groups will be used to perform an exploratory test validation. Each sample will be tested in four different dilutions. For each dilution, we are going to record the optical density (OD) value as raw data. The higher the OD value lies, the higher amount of antibodies is present in the serum. For negative samples, i.e. when specific antibodies are not present, the OD value is higher than 0 (background). The OD values are plotted on a graph to generate a curve. The area under the curve (AUC) will be calculated using the R software. The AUC represents the antibody titer. These graphs are examples for the graphs of a seropositive and a seronegative person:

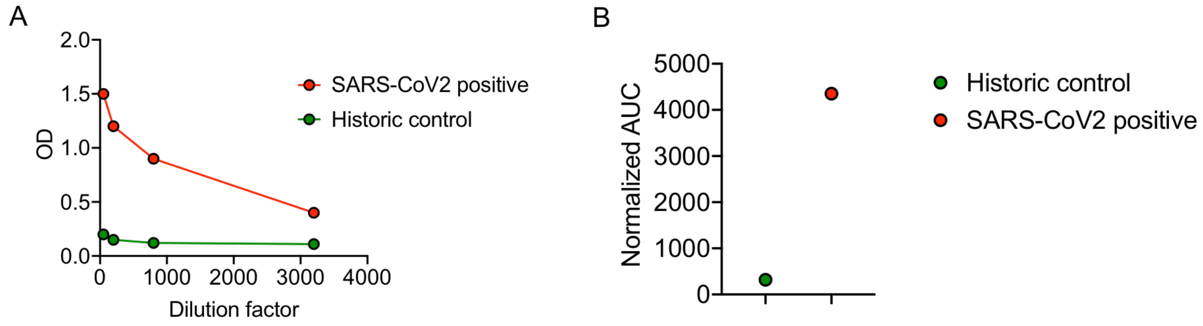

We assume that there is a variability in the AUC for seropositive individuals and historic controls. Within the explorative preliminary study, we will use the historic controls and the individuals with diagnosed SARS-CoV2 infection to look for a cut-off for the AUC. This means that the AUC value is not determined before the start of the trial but will be calculated based on the results of the study participants. Our aim is to develop an antibody test that allows to separate seropositive from seronegative individuals.

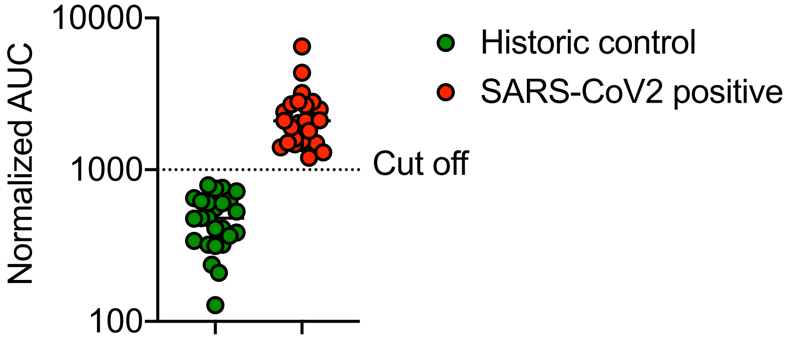

This cut off will be used to study the rest of the population.
We are going to calculate the percentage of the study population that has an antibody titer higher than the cut-off value as well as the transmission rate in households with at least one seropositive individual. Using a standardized questionnaire, we are going to collect data on age, sex, symptoms and exposure. Furthermore, we will perform investigations on virus-specific T cells and metabolic parameters. Using ANOVA, we are going to compare these parameters between seronegative subject, seropositive asymptomatic subjects and seropositive symptomatic subjects.

11. Data management

All personal information of study participants will be collected in a pseudonymized manner. All data which allow identification of the individual (name, date of birth) will be replaced by a pseudonym (e.g. a number, so called Subject-ID). These data will be stored and analyzed electronically. A list with all Subject-IDs will be generated. The name, date of birth and sex will be stored. Individuals belonging to the same household will be grouped together. This Subject-ID list will be available only to the principal investigator, Prof. Dr. C. Waller and the Deputy Researcher, Dr. P. Apostolova. These individuals must keep the data confidential. All data protection requirements are fulfilled. These data are protected from unauthorized access and will not be provided to a third party
Scientific publications will be provided in an anonymized manner that does not allow identification of a single individual.
If applicable, the results from the experiments will be provided to the respective Occupational Medicine physician after the study participants have given their consent. All personal information will be anonymized after the scientific project is completed. No later than 10 years after the project has been completed, these data will be erased.

A revocation of the informed consent is possible at any time point without having to provide specific reasons. If an individual terminates their participation, no further data will be collected. The data that have already been collected will be anonymized so that no identification of the person is possible.

The samples will be collected and initially processed at the Medical Center - University of Freiburg. For some experiments, the samples will be transferred to the Max-Planck Institute for Immunobiology and Epigenetics. This will occur in a pseudonymized manner, so that no identification of the personal identity of the participants is possible. All remaining biological material will be disposed of at the end of the trial.
