# Supplementary material for: IL-33 expression in response to SARS-CoV-2 correlates with seropositivity in COVID-19 convalescent individuals
Source: Nat Commun. 2021 Apr 9;12:2133. doi: 10.1038/s41467-021-22449-w (PMC8035172; doi:10.1038/s41467-021-22449-w)
Supplement: Supplementary file 2 — Reporting Summary [file 41467_2021_22449_MOESM2_ESM.pdf]

nature research

Corresponding author(s): Erika L Pearce

Last updated by author(s): Feb 26, 2021

Reporting Summary

Nature Research wishes to improve the reproducibility of the work that we publish. This form provides structure for consistency and transparency in reporting. For further information on Nature Research policies, see our Editorial Policies and the Editorial Policy Checklist.

Statistics

For all statistical analyses, confirm that the following items are present in the figure legend, table legend, main text, or Methods section.

n/a	Confirmed	
☐	☒	The exact sample size (*n*) for each experimental group/condition, given as a discrete number and unit of measurement
☐	☒	A statement on whether measurements were taken from distinct samples or whether the same sample was measured repeatedly
☐	☒	The statistical test(s) used AND whether they are one- or two-sided<br>*Only common tests should be described solely by name; describe more complex techniques in the Methods section.*
☐	☒	A description of all covariates tested
☐	☒	A description of any assumptions or corrections, such as tests of normality and adjustment for multiple comparisons
☐	☒	A full description of the statistical parameters including central tendency (e.g. means) or other basic estimates (e.g. regression coefficient) AND variation (e.g. standard deviation) or associated estimates of uncertainty (e.g. confidence intervals)
☐	☒	For null hypothesis testing, the test statistic (e.g. F, t, r) with confidence intervals, effect sizes, degrees of freedom and P value noted<br>*Give P values as exact values whenever suitable.*
☒	☐	For Bayesian analysis, information on the choice of priors and Markov chain Monte Carlo settings
☐	☒	For hierarchical and complex designs, identification of the appropriate level for tests and full reporting of outcomes
☐	☒	Estimates of effect sizes (e.g. Cohen's *d*, Pearson's *r*), indicating how they were calculated

Our web collection on statistics for biologists contains articles on many of the points above.

Software and code

Policy information about availability of computer code

Data collection	No specilized softward or code was used for data collection.
Data analysis	Clinical data, ELISA readings, flow cytometry results and single cell RNA sequencing data were processed and analyzed using R (Lucent Technologies version 4.0), which was also employed to generate graphs. R packages used: patchwork (v.1.1.0), reshape2 (v1.4.4), dplyr (v. 0.8.5), CytoExploreR (v. 1.0.8), pheatmap (v. 1.0.12), uwot (v. 0.1.10) and ggplot2 (v. 3.3.2). Flow cytometry data was also analysed using FlowJo (v. 10.7) and FACS Diva (v. 9.0).

For manuscripts utilizing custom algorithms or software that are central to the research but not yet described in published literature, software must be made available to editors and reviewers. We strongly encourage code deposition in a community repository (e.g. GitHub). See the Nature Research guidelines for submitting code & software for further information.

Data

Policy information about availability of data

All manuscripts must include a data availability statement. This statement should provide the following information, where applicable:
- Accession codes, unique identifiers, or web links for publicly available datasets
- A list of figures that have associated raw data
- A description of any restrictions on data availability

All data reported in the current study has been submitted to the publisher and is available for download. Source data are provided with this paper in a single excel file where all the clinical characteristics and measured variables are recorded per patient. Data associated with Fig. 1 and Extended Data Fig. 1 are in the first sheet. Data associated with Fig. 2 and Extended Data Fig. 3 are in the second sheet. Data associated with Fig. 3 and Extended Data Fig. 4 are in the third sheet. There are no restrictions in the use of these data.

Field-specific reporting

Please select the one below that is the best fit for your research. If you are not sure, read the appropriate sections before making your selection.

- [x] Life sciences
- [ ] Behavioural & social sciences
- [ ] Ecological, evolutionary & environmental sciences

For a reference copy of the document with all sections, see nature.com/documents/nr-reporting-summary-flat.pdf

Life sciences study design

All studies must disclose on these points even when the disclosure is negative.

Sample size	SARS-CoV-2-exposed subjects (n=155) were recruited with a standardized procedure based on assessment of inclusion and exclusion criteria. Inclusion criteria were contact to SARS-CoV-2-infected individuals, age of at least 5 years and the ability to provide a written informed consent. The exclusion criterium was the presence of symptoms of an acute SARS-CoV-2 infection (fever, cough, malaise) within the last 14 days prior to blood collection. Study participants gave their written informed consent prior to study enrollment. For subjects <18 years of age, written informed consent was provided by one parent. Sample size was not determined using statical criteria, rather it was a result of the nature of the study. This is a monocentric observational exploratory study of individuals professionally exposed to SARS-CoV-2 and their families.
Data exclusions	No data was excluded.
Replication	Internal controls were included in all experiments to guarantee technical quality of techniques used. Stimulation experiments were repeated 3-4 times. All attempts at replication were successful. Serum measurements were performed only once in each participant, as individual samples were measured at multiple dilution factors, and we observed in all cases that signals were dependent on the sample concentration, thus increasing confidence in the result.
Randomization	Samples were processed in batches as they were collected. No preference was given to serological groups (as this was unknown during sample collection). Because this was an observational study, there was no subject allocation to any experimental intervention groups that would require randomization.
Blinding	Data collection, in vitro experiments and data analysis were all performed blind, with serological groups identifiable only after data analysis during the visualization stage. Serological status and infection history of subjects was not known to scientists collecting samples or performing measurements.

Reporting for specific materials, systems and methods

We require information from authors about some types of materials, experimental systems and methods used in many studies. Here, indicate whether each material, system or method listed is relevant to your study. If you are not sure if a list item applies to your research, read the appropriate section before selecting a response.

Materials & experimental systems

n/a	Involved in the study	
	x	Antibodies
	x	Eukaryotic cell lines
x		Palaeontology and archaeology
x		Animals and other organisms
	x	Human research participants
	x	Clinical data
x		Dual use research of concern

Methods

n/a	Involved in the study	
x		ChIP-seq
	x	Flow cytometry
x		MRI-based neuroimaging

Antibodies

Antibodies used	All antibodies were used at a dilution of 1:200, unless otherwise specified:<br>AlexaFluor647 anti-human CD3 (Biolegend, Clone: HIT3a, Cat#300322)<br>PE anti-human CD3 (Biolegend, Clone: HIT3a, Cat# 300308)<br>BrilliantViolet 711 anti-human CD4 (Biolegend, Clone: OKT4, Cat#317440)<br>FITC anti-human CD4 (Biolegend, Clone: A161A1, Cat# 357406)<br>Pacific Blue anti-human CD8 (Biolegend, Clone: HIT8a, Cat#300928)<br>APC anti-human CD8 (Biolegend, Clone: HIT8a, Cat# 300812)<br>BrilliantViolet 510 anti-human CD14 (Biolegend, Clone: M5E2, Cat# 301842)<br>APC-Cy7 anti-human CD14 (Biolegend, Clone: M5E2, Cat# 301820)<br>BrilliantViolet 605 anti-human CD16 (Biolegend, Clone: 3G8, Cat# 302039)<br>APC-Cy7 anti-human CD16 (Biolegend, Clone: 3G8, Cat# 302017)

PE anti-human CD19 (Biolegend, Clone: HIB19, Cat# 302208)
APC-Cy7 anti-human CD19 (Biolegend, Clone: HIB19, Cat# 302217)
PE/Dazzle 594 anti-human CD27 (Biolegend, Clone: M-T271, Cat# 356421)
PE-Cy7 anti-human CD45RA (Biolegend, Clone: HI100, Cat# 304112)
APC-Cy7 anti-human CD56 (Biolegend, Clone: HCD56, Cat# 318332)
AlexaFluor 488 anti-human CD62L (Biolegend, Clone: DREG56, Cat# 304816)
BrilliantViolet 785 anti-human CD69 (Biolegend, Clone: FN50, Cat# 310932)
PE/Dazzle 594 anti-human CD134 (Biolegend, Cline: ACT35, Cat# 350019)
BrilliantViolet 785 anti-human CD197 (Biolegend, Clone: G043H7, Cat# 353230)
AlexaFluor 488 anti-human FOXP3 (Biolegend, Clone: 206D, Cat# 320111)
AlexaFluor647 anti-human IFNg (Biolegend, Clone: 4SB3, Cat# 502516)
AlexaFluor700 anti-human TNF (Biolegend, Clone:MAb11, Cat# 502928)
PE-Cy7 anti-human TNF (eBioscience, Clone:TN3-19.12, Cat# 25-7423-82)
FITC anti-human IL-1 beta (eBioscience, Clone: CRM56, Cat# 11-7018-42)
eFluor 450 anti-human IL-6 (eBioscience, Clone: MQ2-13A5, Cat#48-7069-42)
APC anti-human IL-10 (Biolegend, Clone: JES3-9D7, Cat# 501409)
Alexa Fluor 488 anti-human CD11c (Biolegend, Clone: 3.9, Cat# 301618)
Alexa Fluor 647 anti-human CD185 (Biolegend, Clone:J252D4, Cat# 356906)
PE-Cy7 anti-human IgD (Biolegend, Clone: IA6-2, Cat# 348210)
PE-Cy7 anti-human CD33 (Biolegend, Clone: P67.6, Cat# 366617)
Brilliant Violet 785 anti-human CD278 (ICOS) (Biolegend, Clone: C398.4A, Cat# 313533)
PE anti-human PD-1 (Biolegend, Clone: EH12.2H7, Cat# 329905)
FITC anti-human ST2L (mdbiosciene, Clone: B4E6, Cat# 101002F)
Brilliant Violet 650 anti-human CD38 antibody (Biolegend, Clone: HB-7, Cat# 356619)
PE recombinant anti-IL-33 antibody (Abcam, Clone: 002, Cat# ab275607), dilution 1:500
PE recombinant Rabbit IgG (Abcam, Clone: EPR25A, Cat# ab209478)
IL-33 monoclonal antibody (Invitrogen, Clone: 6H617, Cat# MA5-16242), dilution 1:500
PE rat anti-mouse IgG1 (BD Pharmingen, Clone: A85-1, Cat# 550083)
Goat anti-human IgG (H+L), secondary antibody, HRP-conjugated (ThermoFisher Scientific, Cat#31410)
Anti-human IgM (μ-chain specific)-Peroxidase antibody (Sigma Aldrich, Cat#A0420)

Validation

All antibodies were commercial in source and as such validated by the manufacturer.
All flow cytometry antibodies from Biolegend and eBioscience were subjected to standard procedures for flow cytometry validation by the manufacturers.
All newly developed clones at BioLegend undergo validation testing for multiple applications. This serves as a cross-check for specificity and provides clarity for research uses. Typically, antibodies are tested by two or more of the below methods (flow cytometry, western blot, chromatin immunoprecipitation, immunofluorescence, immunohistochemistry, biofunctional assays). Thus, the clone cross-validates itself, by demonstrating functionality across orthogonal testing methods. Additionally, the biological induction of the expression further validates the specificity of the antibody. Knockout or knockdown of gene expression, such as with siRNA, is also an excellent tool for target validation.
More information regarding validation and reproducibility can be found online on the manufacturer's website:
https://www.biolegend.com/en-us/reproducibility
https://www.thermofisher.com/antibody/product/IL-1-beta-Antibody-clone-CRM56-Monoclonal/11-7018-42
https://www.thermofisher.com/antibody/product/TNF-alpha-Antibody-clone-TN3-19-12-Monoclonal/25-7423-82
https://www.thermofisher.com/antibody/product/IL-6-Antibody-clone-MQ2-13A5-Monoclonal/48-7069-42

The IL-33 Antibody (clone: MA5-16242) was validated by the manufacturer for flow cytometry and western blot by staining of Jurkat cells, HUVEC cells and with a partial recombinant IL-33 control. The PE-conjugated monoclonal anti-IL-33 antibody (Clone 002, Abcam) has been validated with the Abpromise guarantee approach of the manufacturer for use in flow cytometry with HUVEC cells used as a positive control.
Abcam validation statement: Reproducibility is key to advancing scientific discovery and accelerating scientists' next breakthrough. Abcam is leading the way with our range of recombinant antibodies, knockout-validated antibodies and knockout cell lines, all of which support improved reproducibility. We are also planning to innovate the way in which we present recommended applications and species on our product datasheets, so that only applications & species that have been tested in our own labs, our suppliers or by selected trusted collaborators are covered by our Abpromise™ guarantee. In preparation for this, we have started to update the applications & species that this product is Abpromise guaranteed for. We are also updating the applications & species that this product has been "predicted to work with," however this information is not covered by our Abpromise guarantee. Applications & species from publications and Abreviews that have not been tested in our own labs or in those of our suppliers are not covered by the Abpromise guarantee.
Further information on validation can be found on the manufacturers' websites:
https://www.abcam.com/pe-il33-antibody-002-ab275607.html
https://www.thermofisher.com/antibody/product/IL-33-Antibody-clone-6H617-Monoclonal/MA5-16242

Eukaryotic cell lines

Policy information about cell lines

Cell line source(s)

Expi293F cells were purchased from ThermoFisher Scientific.

Authentication	No further authentification was performed.
Mycoplasma contamination	No mycoplasma contamination was found.
Commonly misidentified lines (See ICLAC register)	No commonly misidentified cell line was used in this study.

Human research participants

Policy information about studies involving human research participants

Population characteristics	SARS-CoV2 pre-exposure donor serum samples (n=16) had been collected, aliquoted and stored at the Medical Center - University of Freiburg prior to June 1st, 2019. These individuals were considered to be pre-exposure controls since their biological material had been stored prior to the emergence of SARS-CoV-2. This cohort included 50% males (n=8) and 50% females. Median age was 51 years with a range of 39–77 years.<br>SARS-CoV-2-exposed subjects (n=155) were recruited with a standardized procedure based on assessment of inclusion and exclusion criteria. Inclusion criteria were contact to SARS-CoV-2-infected individuals, age of at least 5 years and the ability to provide a written informed consent. The exclusion criterium was the presence of symptoms of an acute SARS-CoV-2 infection (fever, cough, malaise) within the last 14 days prior to blood collection.
Recruitment	SARS-CoV-2-exposed subjects (n=155) were recruited with a standardized procedure based on assessment of inclusion and exclusion criteria. Inclusion criteria were contact to SARS-CoV-2-infected individuals, age of at least 5 years and the ability to provide a written informed consent. The exclusion criterium was the presence of symptoms of an acute SARS-CoV-2 infection (fever, cough, malaise) within the last 14 days prior to blood collection. These individuals were recruited based on existing SARS-CoV-2 exposure and signing of a written informed consent without selection for any other than the mentioned criteria. Due to the nature of this study, the seropositivity rate for SARS-CoV-2 in the study population was higher than in the general population at this time point.<br>SARS-CoV-2 pre-exposure control subjects were enrolled during presentations for a regular examination at the Department of Medicine at the Medical Center - University of Freiburg. Their biomaterial was collected after written informed consent for biobank storage purposes. At the time point of this trial, no information other than sex and age was available for these subjects, so that self-selection bias did not interfere with the examinations in this study.
Ethics oversight	Ethics Committe of the Medical Center - University of Freiburg (approval number 305/20)

Note that full information on the approval of the study protocol must also be provided in the manuscript.

Clinical data

Policy information about clinical studies

All manuscripts should comply with the ICMJEguidelines for publication of clinical research and a completedCONSORT checklist must be included with all submissions.

Clinical trial registration	The study was registered at the German Clinical Trial Register (DRKS00022292).
Study protocol	The full trial protocol has been submitted with the revised version of the manuscript.
Data collection	SARS-CoV2 pre-exposure control samples were collected prior to 01.06.2019. SARS-CoV2 exposed subject material was collected either from biobank storage (collection date between 01.04.2020 and 31.07.2020) or from newly recruited participants (starting from 01.07.2020). All samples were collected at the Medical Center - University of Freiburg. Experimental data was collected between 01.05.2020 and 15.01.2021.
Outcomes	This is an observational study where certain parameters of scientific interest were defined and measured. These include the anti-Spike, anti-RBD IgG and IgM antibody titers, percentage of different immune cell populations, production of inflammatory cytokines and expression of IL-33 and ST2L on cells. These endpoints were measured by ELISA or flow cytometry.

Flow Cytometry

Plots

Confirm that:

- [x] The axis labels state the marker and fluorochrome used (e.g. CD4-FITC).
- [x] The axis scales are clearly visible. Include numbers along axes only for bottom left plot of group (a 'group' is an analysis of identical markers).
- [x] All plots are contour plots with outliers or pseudocolor plots.
- [x] A numerical value for number of cells or percentage (with statistics) is provided.

Methodology

Sample preparation	PBMCs were isolated from freshly isolated full blood by density centrifugation using SepMate-50 PBMC isolation tubes and Lymphoprep density gradient medium. After isolation, PBMCs were frozen in 90% FCS, 10% DMSO and stored in liquid nitrogen until the time of analysis.

Instrument	BD FACSymphony A5, BD LSR Fortessa
Software	Data were collected using FACSDiva 9.0 and analyzed using FlowJo 10.7.
Cell population abundance	No cell sorting has been performed in this study.
Gating strategy	For all flow cytometry experiments, the lymphocyte cell population was gated first based on FSC-A and SSC-A. To include only single cells, we performed gating based on FSC-A and FSC-H. Dead cells were excluded by gating on the negative fraction after staining with Live/Dead Fixable dyes. The populations of interest were selected by using combination of the respective markers, for example CD45, CD4, CD8, CD19 and others. Detailed gating strategies for all cell populations described in this paper are provided in Extended Data Figure 2.

☒ Tick this box to confirm that a figure exemplifying the gating strategy is provided in the Supplementary Information.
